# Extraction and Modification of Macroalgal Polysaccharides for Current and Next-Generation Applications

**DOI:** 10.3390/molecules25040930

**Published:** 2020-02-19

**Authors:** Madeleine Jönsson, Leila Allahgholi, Roya R.R. Sardari, Guðmundur O. Hreggviðsson, Eva Nordberg Karlsson

**Affiliations:** 1 Biotechnology, Department of Chemistry, Lund University, Post Office Box 124, 221 00 Lund, Sweden; madeleine.jonsson@biotek.lu.se (M.J.); leila.allahgholi@biotek.lu.se (L.A.);; 2 Faculty of Life and Environmental Sciences, University of Iceland, Askja, IS-101 Reykjavík, Iceland; gudmundo@matis.is; 3 Matis Ohf, Vinlandsleid 12, IS-113 Reykjavik, Iceland

**Keywords:** seaweed, composition, structure, extraction, enzymatic modification, application, bioactivity

## Abstract

Marine macroalgal (seaweed) polysaccharides are highly promising for next-generation applications in several industries. However, despite the reported comprehensive potential of these polysaccharides, commercial products are scarce on the market. Seaweed cultivations are increasing in number and production quantity, owing to an elevated global trend of utilization interest in seaweed. The extraction of polysaccharides from seaweed generally generates low yields, but novel methods are being developed to facilitate and improve the extraction processes. Current areas of applications for seaweed polysaccharides mainly take advantage of the physicochemical properties of certain polysaccharides, such as gelling, thickening and emulsifying. However, many of the numerous bioactivities reported are still only at research level and lack clinical evidence for commercialization. It has been suggested the construction of smaller units may generate better defined molecules that are more suitable for biomedical applications. Enzymatic modification is a promising tool for the generation of more defined, targeted biomolecules. This review covers; structural differences between the most predominant marine algal polysaccharides, extraction processes, modification alternatives, as well as a summary of current and potential next-generation application areas.

## 1. Introduction

Marine macroalgae (seaweeds) are highly promising as biomass resources for next generation biorefineries. Seaweeds include several species of macroscopic, multicellular marine algae. According to Chapman and Chapman [1], “Seaweeds belong to a rather ill-defined assemblage of plants known as the algae. The term ‘seaweed’ itself does not have any taxonomic value, but is rather a popular term used to describe the common large attached marine algae found in the groups Chlorophyceae, Rhodophyceae, Phaeophyceae or green, red and brown algae respectively”.

Currently, approximately 32 million tonnes (wet weight) of seaweed is harvested globally [2], of which around one million tonnes are harvested wild, while the harvest of the remaining 31 million tonnes involves farmed seaweeds—with the largest production countries being China, Indonesia and the Philippines [2]. In Europe, wild harvests still dominate, with Norway being one of the leading producers globally (0.16 million tonnes harvested wild in 2016) [2]. Interest in the cultivation of seaweeds in Europe is however growing, although it is still at an early stage, with a total production of less than 2000 tonnes in 2016 (mainly large brown kelps, such as *Saccharina latissima*, *Undaria pinnatifida*, and *Alaria esculenta*) [3]. The most important farmed seaweed species include the red seaweeds *Eucheuma* spp. (10.2 million tonnes), *Gracilaria* spp. (3.9 million tonnes), *Kappaphycus* spp. (1.8 million tonnes) and *Porphyra* spp. (also called nori, 1.2 million tonnes), and the brown seaweeds *Saccarina japonica* (brown seaweed species, 8.0 million tonnes) and *U. pinnatifida* (also called wakame, 2.3 million tonnes) [4].

Seaweed cultivation initiatives in Europe are driven both by the demand for sustainable biomass resources for industrial applications, and the increasing attention on more sustainable food production and consumption. Seaweeds are promising in these aspects, due to their high productivity (exceeding terrestrial biomass), the possibilities of bulk-scale farming without the need for fertilizers (not competing with agricultural land), combined with causing very limited harm to the seabed or to fishery resources [4,5,6]. Seaweed farming requires no fresh water for cultivation and occupies no terrestrial space. In addition, seaweeds have a high carbohydrate content (approximately 50% DW (dry weight) in different polymeric forms [5,6]) and they contain essential amino acids [7]. Seaweeds are also known to contain various compounds with bioactivity, including a number of their polysaccharides, which have been investigated in numerous research articles, but are not yet established as industrial products.

The main components in seaweeds include: polysaccharides, minerals, proteins, small amounts of lipids, polyphenols and pigments. The seaweed polysaccharides pose a challenge as industrial feedstock because of their structural complexity, “unconventional” and heterogeneous sugar composition, sulfation and other modifications. The possible product range from macroalgae may, however, surpass other biomass of comparable bulk and ease of cultivation. Besides all lignocellulose sugars, hexoses (glucose, mannose and galactose), and pentoses (xylose and arabinose), appreciable amounts of “rare sugars” (sugar alcohols, deoxy sugars and sugar acids (uronates)) are present in macroalgae. These recalcitrant macroalgal polysaccharides are relatively new to industry, but they need to be utilized to ensure full valorisation of seaweed biomass. Added value derivatives that can be obtained by enzymatic and microbial conversions include food and feed additives, such as modified polysaccharides and oligosaccharides with reported health promoting effects. However, the most attractive unrealized potential may be in unique, speciality chemicals and platform chemicals, based on properties and possibilities due to their rare sugars, for the chemical and pharmaceutical industries.

The combined potential of underexploited biomass, the prospect of vastly increased global production, novel added-value products, and the anticipated expanded product range from seaweed biomass has led to a number of European projects involving both seaweed cultivation and the development of technological platforms for fractionation, extraction and pre-processing. Continued work in this field will help to overcome current challenges in terms of limited shelf-life of the harvested material, and further developments in establishing biocatalytic refining processes will help to expand the product portfolios that today are mainly confined to purified components from the biomass [8]. The main products of seaweeds are still the carbohydrate polymers alginate (brown seaweeds), agar and carrageenan (red seaweeds), which are extracted and refined in bulk and have important uses as hydrocolloids, emulsifiers and thickeners [1]. These compounds are produced by methodologies that can be described as mechanical and/or physicochemical extraction and fractionation technology, and hence they are basically unmodified seaweed components. Products (on a more industrial scale) based on biocatalytic or microbial conversions of seaweed biomass are, in principle, limited to conversion to energy via anaerobic digestion, which still has some challenges to overcome to become economically viable [9]. Enzyme conversions are still mostly at research level [8] but hold a range of possibilities as the necessary processes and tools are being developed at this stage.

In this review, we outline the main components of marine macroalgae, including brown, red, as well as green seaweed species. Different protocols to extract and fractionate defined components are shown, and modification possibilities of purified extracts are mentioned. Application areas of seaweeds are also presented, showing both currently existing as well as novel possibilities.

## 2. Brown Seaweed Polysaccharides

Brown seaweed, Phaeophyceae, are widespread macro algae growing abundantly at northern latitudes in marine cold waters [10], although some species are indigenous to fresh and brackish waters. Brown algae contain a carotenoid pigment, fucoxanthin, providing the plant’s characteristic brown color by suppressing other pigments. The sizes of brown algae plant bodies range from a few millimeters up to some 70 m [11], indicating a variety in structure and composition between species within the same algal class.

Brown macroalgae consist of a complex and dynamic cell wall rich in polysaccharides, such as alginate (also alginic acid and algin), fucoidan (also fucoidin and fucan), laminarin (also laminaran), and cellulose [12,13], but also containing polyphenols, proteins, glycoproteins, phlorotannins (sulfated phenolic compounds), halogens such as iodine, and minerals, such as sodium, potassium, calcium and magnesium [11,13]. The relative content of different compounds varies between species and is highly dependent on seasonal, environmental and regional factors [14]. Additionally, while alginate seems to constitute a significant part of all brown algae, laminarin and fucoidan appear to occur more frequently in some species, such as *Laminaria* spp. and *Fucus* spp., than in others [11]. The presence of free sugars, such as glucose, fructose and sucrose in brown algae is insignificant, since monomeric sugars are immediately converted into d-mannitol and polysaccharides [14]. Glucose content, for instance, is seasonally dependent, bound mainly in the beta-glucan, laminarin that acts as storage polysaccharide in the cells.

Deniaud-Bouët et al. [12] suggest that the cell wall of brown algae consists of fucose containing sulfated polysaccharides (FCSPs) as a major crosslinking glycan in the cell walls, interlocking the cellulose microfibrils situated in layers parallel to the cell surface that constitute the framework of the cell. They also suggest that short fragments of hemicellulose are intermediate links between cellulose and the cross-linking FCSPs, although the composition of these proposed hemicellulose fragments is not defined. These structures are embedded in a matrix of alginates. The cell wall matrix is postulated to consist of mainly alginates, proteins, polyphenols (linked to both these compound groups), and iodine. Cellulose and/or mixed linkage glucans, comprising only a minor fraction (<10% DW) [15], may also have a structural role in the cell wall as microfibrils associated with fucoidan or alginate [12,16].

Brown algae appears to have emerged later in time than red algae, green algae and terrestrial plants. No unicellular brown algae species have been found, and the development of the multicellularity and plant-like structures of brown algae occurred isolated from other algae and plants. However, brown algae have cell wall polysaccharides in common with plants (cellulose), animals (sulfated fucans) and bacteria (alginate) [13]. Although brown algae comprise a wide variety of compounds, most focus and research has been directed towards the utilization of algal polysaccharides, especially fucoidan, alginate and laminarin.

### 2.1. Fucoidan

Fucoidans are categorized as fucose-containing sulfated polysaccharides (FCSPs), which encompass the polysaccharides of various chain lengths, structures of branching, and degrees of sulfation. The predominant component in fucoidan is fucose, but it also contains other monomers such as galactose, mannose, xylose and residues of glucuronic acid [17,18]. Fucoidans are divided into two subgroups; one group whose backbone structure constitutes α-1,3-l-fucopyranose residues, while the second group is composed of alternating 1,3- and 1,4-linked α-l-fucopyranose residues [19] (Table 1). The variation between fucoidans is reflected in the vast divergences in reported molecular weights; from about 40 kDa up to 1600 kDa [20].

The fucoidan content appears to be higher during autumn than at any other time of the year [11,20]. Fucoidans are considered important components for regulating water and ion retention in extracellular matrixes of the plant, to prevent desiccation and osmotic stress when exposed to low tides [13]. It is unclear whether composition alterations correlate only with an increase in storage polysaccharides or if the changes impact plant tissue structure as well [21].

### 2.2. Alginate

Alginates are the salts of alginic acid, which are linear unbranched copolymers comprised of β-1,4-d-mannuronic acid (M) and α-1,4-l-guluronic acid (G) (Table 1). The size of the copolymer, M/G-ratio and the properties of integrated G-blocks affects the functions, physical properties, mechanical strength and biocompatibility of the compound [28,29]. The M/G-ratio of alginates varies between algal species, harvest period, as well as between the structure and age of the algae tissue. The chelating properties of alginate are dependent on the length of the compounds’ G-blocks (repetitive structure of guluronic acid) [30]. Due to the rheological properties of alginates, these polysaccharides are widely used in industries such as the pharmaceutical, medical technology, cosmetic, food, agricultural, textile and paper industries [29].

Alginates are mainly used industrially for their physical characteristics such as their stabilizing, thickening and emulsifying properties, but depending on specific properties, such as gel strength, porosity or biocompatibility, they are expanding into applications such as biomaterials for tissue engineering and bioprinting [31]. Alginates are analogues to pectin in terrestrial plants [11]. The current and potential application of alginates will be further appraised in Section 7.

Alginates extracted from brown algae are categorized as phycocolloids, polysaccharides able to form colloidal systems when dispersed in water [11]. Alginates are formed when alginic acid interacts with present metal ions such as calcium, sodium or magnesium. Calcium alginates form gels which are insoluble in water, while sodium and magnesium salts of alginic acid turn into water-soluble gels [32]. The glycosidic bonds between sugar monomers of alginate polymers can be exposed to cleavage in both acidic and alkalic conditions, which causes degradation of the compound. Other factors affecting the stability of the copolymer are temperature and free radicals generated by contaminants such as polyphenols [29].

### 2.3. Laminarin

Laminarin is a β-1,3-glucan, consisting of β-1,3-d-glucopyranose units interspersed with β-1,6-linked d-glucopyranose units forming branch-points or interchain residues (Table 1). It is a comparatively short polymer consisting of about 20–25 monomers [14,33]. Laminarin is the carbohydrate reserve of many brown seaweeds, corresponding to starch (α-1,4-glucans) in terrestrial plants [10,14,34]. Interchain β-1,6-linkages contribute to the branching structure of laminarin and determine the water solubility of the compound. Increased branching is associated with elevated solubility in water [14]. Two different types of laminarin, G-type and M-type, have been explored, and they simply differ in the reducing end of the polymer chain. Whilst laminarin G-type only contains glucopyranose residues, the M-type is terminated with 1-*O*-linked d-mannitol [14,34]. The ratio between M-type and G-type chains can be as high as 3:1 as observed in *Laminaria digitata* [35], while M-types were reported to be absent in laminarin from *Eisenia bicyclis* [36].

Laminarin, and laminarin-derived oligosaccharides, can be expected to elicit the same health-promoting responses in organisms as reported for other β-glucans, including prebiotic, antioxidant, anticoagulant, hypocholesterolemic, and antimutagenic activity, and additionally, they have reported anti-inflammatory and anti-cancer effects that appear to be confined to β-1,3-(1,6)-glucans [37]. Immunomodulating activities vary between laminarins (Hreggviðsson and Nordberg Karlsson, unpublished), depending on species, and may be related to varying content, type (branchpoints or interchain) and spatial distribution of the β-1,6-linkages. The ratio between β-1,6- and β-1,3-linkages in laminarin tends to vary between species; from a very low ratio in laminarin from *Laminaria hyperborea,* which is composed mainly of β-1,3-linkages [38], to a moderate ratio of 1:7 in laminarin from *L. digitata*, and to a high range of 1:3 or 2:3 in laminarin from *E. bicyclis* [35,36].

## 3. Red Seaweed Polysaccharides

Red seaweed, Rhodophyta, is a group of mainly multicellular photosynthetic eukaryotic organisms, reported to comprise more than 7000 species. They grow mostly in marine habitats, but up to 5% of species can grow in freshwater environments, with greater numbers in warmer areas. The red color is expressed due to the predominance of the pigments phycocyanin and phycoerythrin over other pigments such as chlorophyll A and carotene. Red algae store sugars as floridean starch, a highly branched amylopectin without amylose, which is accumulated outside the plastid in the cytosol of the cells [39]. The cell wall of the seaweed is mainly composed of cellulose, carrageenan and agar, which all have several industrial applications.

### 3.1. Carrageenan

Carrageenan is a high molecular weight sulfated polysaccharide, available in the cell wall of red seaweed interacting with other bioactive compounds including proteins, lipids, pigments and other polysaccharides. It is composed of alternating α-1,3-linked d-galactopyranosyl and β-1,4-linked d-galactopyranosyl groups and 3,6-anhydrogalactose residues.

The three most important commercial types of carrageenan are kappa (κ), iota (ι) and lambda (λ) carrageenan (Table 1), which differ in the amount and position of sulfate ester groups and the number of 3,6-anhydrogalactose residues. κ-carrageenan is composed of 25–30% sulfate ester groups and 28–35% anhydrogalactose. The sulfate ester content in ι–carrageenan is similar to that of κ-carrageenan (28–30%), but it contains a lower fraction of anhydrogalactose units (25–30%). The sulfate substitution in λ-carrageenan is comparatively high (32–39%), but it contains no anhydrogalactose residues [24]. The backbone can be substituted by pyruvate ketal, which is only reported for the λ-family of carrageenans [40].

The composition of carrageenan varies between species. For example, carrageenan from *Kappaphycus alvarezii* (in the trade also called *Eucheuma cottonii*) is made of mainly κ-carrageenan, whereas *Eucheuma dendiculatum* (trade name *Eucheuma spinosum*) consists of ι-carrageenan. *Chondrus crispus* and *Sarcothalia crispata* contain both κ- and λ-carrageenan. The water solubility and gelation of carrageenan depends on the amount of sulfate ester groups and associated divalent cations, such as sodium and potassium, although λ-carrageenan can form a gel in the presence of trivalent ions [41]. Higher numbers of sulfate ester groups result in lower temperatures required for solubility and lower gel strengths, which affects the application of carrageenan [42]. Further information about the source, structure and properties of carrageenan are explained in a comprehensive review by Campo et al. [24]

### 3.2. Agar

Agar is a mixture of polysaccharides with gelling capacity, such as agarose and agaropectin, extracted mostly from *Gelidium* and *Gracilaria* species [43]. Agarose is the major fraction of agar, estimated to constitute 70% of agar polysaccharides. It is a high molecular weight polysaccharide consisting of d-galactose and 3,6-anhydro-α-l-galactose units joined by β-1,3 and α-1,4 glycoside linkages (Table 1) [26,44]. The agar backbone can be substituted with sulfate esters, methoxyl groups and pyruvate ketals [44]. Agaropectin has a lower molecular weight and a higher amount of sulfate ester groups than agarose. It has the same backbone as agarose, which, in agaropectin, is substituted with various amounts of sulfate esters, d-guluronic acids and pyruvic acids.

## 4. Green Seaweed Polysaccharides

Green seaweeds are members of the Chlorophyta, which include the majority of the described species of green algae [45,46]. Members of the Chlorophyta are inhabitants of both marine and freshwater systems. Marine macroalgae (the focus in this review) from the classes of Chlorophyta in principle involve the class Ulvophyceae [47] and mainly include candidates from the orders Ulvales-Ulotrichales, Cladophorales, and Bryopsidales, including species that display large morphological and cellular diversity [45,48,49]. Well known genera classified under Ulvophyceae include various edible species of *Ulva*, *Gayralia* and *Monostroma* (classified under the order Ulvales-Ulvotrichales), *Cladophora* (Cladophorales), *Caulerpa* and *Codium* (Bryopsidales), all of which have been used in human diets in different parts of the world. Despite this traditional use, green seaweeds are not as well researched as red and brown, and although having potential, they lack major industrial applications, such as those seen for red and brown algae.

Green seaweeds contain interesting polysaccharides that are sulfated and/or carboxylated. The backbones of the main polysaccharides differ in candidate species from different genera, but roughly, they can be divided into two major groups that are classified as uronic acid rich or uronic acid limited [22]. The uronic acid rich (sulfated) polysaccharides are represented by the ulvans that can be found in various species of *Ulva*, *Gayralia* and *Monostroma*. The composition of ulvan is reported to be dependent on species, season, growth conditions, as well as on the method of isolation [27]. Four different types of monosaccharides (rhamnose (Rha), xylose (Xyl), glucuronic acid (GlcA) and iduronic acid (IdoA)) have, along with sulfate groups, been reported in different types of ulvans, which are also described as consisting of several disaccharide fragments. All four types of monosaccharides can occur in the backbone, while only glucuronic acids have, according to Synytsya et al. [22], also been reported as side chains. The repeating bios-unit is often reported to consist of (uronic acid-Rha), such as in *Ulva* sp., where the uronic acid is either glucuronic acid or iduronic acid. Repeating units with alternating (Rha-Glc-Rha-xyl) structures have also been reported from *Ulva pertusa* [50]. The basic structures of common ulvans are depicted in Table 1. The uronic acid limited polysaccharides occur, for instance, in species of *Codium* and are reported as sulfated galactans, arabinopyranans and mannans.

## 5. Extraction of Seaweed Polysaccharides

Since seaweeds encompass high levels of water, extraction of polysaccharides from the biomass should be preceded by water content determination. While traditional methods for determining moisture level, such as thermogravimetric analyses, are primarily employed, new and faster techniques have potential as alternatives. For instance, near-infrared spectroscopy has proved successful for the fast and accurate analysis of water content in corn stover silage [51], and could be a useful tool also for seaweed. The dry weight is further applied as the basis for yield calculations.

Processes for pretreatment and extraction are usually not distinctly separated when isolating polysaccharides from seaweed, and different extraction methods are commonly combined with attempts to increase yields. In this review, pretreatment and extraction refer to different processing steps applied to macroalgae in order to solubilize the polysaccharides into a liquid medium. Pretreatment methods include processes employed for swelling of macroalgal fibers and increasing pore sizes, or reducing sizes of the biomaterial. The pretreatment of macroalgae consists of mainly mechanical (size reduction, washing, beating, and sonication), thermal (microwave, steam explosion, wet oxidation, and plasma-assisted), chemical (acid or alkali, peroxide), and biological pre-treatments [52]. However, other methods such as liquid ammonia pretreatment have been performed as a promising method for lignocellulosic biomass [53], and might also have potential for the pretreatment of macroalgae. Traditionally, chemical extraction methods have predominantly been used to extract polysaccharides from algal biomass, but have recently been accompanied by new techniques with the aims of increasing extraction yield, using fewer solvents, operating at lower temperatures and decreasing the extraction time (Table 2) [54,55].These efforts aim to reduce energy consumption, thereby increasing the sustainability of the method. This review will further cover extraction processes of polysaccharides from brown, red, and green seaweed.

### 5.1. Extraction of Brown Seaweed Polysaccharides

The available literature suggests a comprehensive number of methods for the extraction of polysaccharides from macroalgae. However, the majority of these refer to modifications of existing conventional methods. The development of extraction methods of fucoidan, one of the more difficult polysaccharides to extract in good yield from brown algae, is reviewed by Ale et al. [18] with a historical perspective (1913–1950).

**Figure 1:**
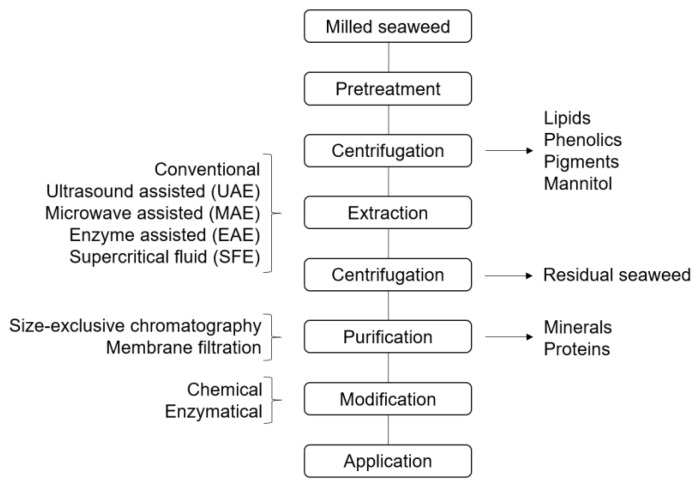
Basic flow chart of extraction of polysaccharides from brown macroalgae.

A general, simplified flow chart of the extraction of brown macroalgal polysaccharides is illustrated in Figure 1. For brown algae, a calcium compound (usually CaCl_2_) can be introduced to the polysaccharide fraction during extraction to precipitate alginate, and thereby separate laminarin from the alginate fraction, while fucoidan is present in various amounts in both fractions [73]. Sulfated polysaccharides in the supernatant are often precipitated with ethanol, and further purification steps—often repeated extractions in acid followed by precipitation, size-exclusive chromatography, or filtration—can be applied to achieve pure fucoidan [59,60,74]. It has been suggested to always include a benchmark procedure when employing new extraction methods for fucoidan, in order to get better comparisons of fucoidan structures [75]. The extraction of laminarin is predominantly performed at increased temperatures, since the water solubility of different forms of laminarin vary. Branched forms tend to be more water soluble than non-branched. The typical extraction yields of laminarin are presented in Table 3. Alginates, being industrially produced from brown macroalgae, are commonly extracted by several ion-exchange procedures using either the ‘acid precipitation method’ or the ‘calcium precipitation method’ [76,77]. Simplified, water-insoluble divalent alginate gel or alginic acid gel are swelled in acidic water prior to extraction. Sodium-containing compounds are then introduced to convert the divalent alginate into water-soluble sodium alginate during extraction. Aqueous alginate is highly viscous and must be diluted prior to clarification and filtration. The typical yield of alginate extraction (Table 3) is usually higher than that of fucoidan and laminarin. However, different isolation methods seem to affect the mannuronic/guluronic acid ratio (M/G ratio) of the extracts differently, which impacts the gelling properties of alginate [78,79].

In Table 3, a selection of different extraction approaches including novel techniques have been compared with respect to their yields. However, although calculated yields are often used as measures of efficacy of the extraction method, the structural integrity of the yielded compound is as important for its function. Additionally, the estimated yield does not take into consideration the variation in polysaccharide content between different species and other external factors, and the purity of the extracted polysaccharide is usually not specified.

### 5.2. Extraction of Red Seaweed Polysaccharides

Carrageenan is extracted from several species of red seaweed including *Hypnea*, *Gigartina*, *Chondrus*, *Eucheuma*, *Kappaphycus*, and *Mastocarpus* [91]. In conventional extraction, an alkali solution is used to extract carrageenan, because in alkaline conditions some of the sulfate ester groups are removed with the formation of 3,6-anhydro-d-galactose units, thus improving gel strength. Extraction parameters such as operation time, temperature, concentration of alkali, and the ratio between the weight of the seaweed and the volume of the alkali solution depend on the seaweed species and the desired quality of extracted carrageenan [25,91,92,93,94].

**Figure 2:**
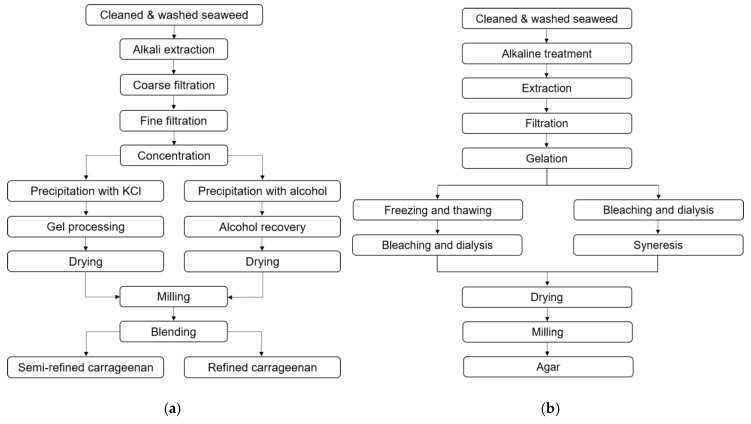
Conventional extraction method of refined carrageenan (RC) and semi-refined carrageenan (SRC) (**a**). Alkaline method for agar extraction [95] (**b**).

There are two conventional extraction procedures to extract refined carrageenan (RC) and semi-refined carrageenan (SRC) (Figure 2a). In RC production, carrageenan is solubilized in hot water containing alkali, such as sodium hydroxide, and the remaining insoluble compounds are separated via filtration. The solution containing carrageenan is concentrated and carrageenan is subsequently precipitated by alcohol, dried and milled.

In SRC production, carrageenan is not solubilized from the seaweed but forms a gel in the presence of potassium chloride. In this method, the seaweed is initially boiled in hot potassium chloride solution. Thereafter, compounds solubilized in the alkali solution including protein, carbohydrate and salts are separated and the remaining seaweed is rinsed to remove residual alkali. The product which looks like seaweed is dried and milled. This method is mainly applied to extract κ-carrageenan, but can also be applied to produce semi-refined ι-carrageenan. The market for κ-carrageenan is significantly higher than for ι-carrageenan due to the favorable properties of κ-carrageenan in many industries [91,95]. SRC is generally cheaper than RC, as no alcohol precipitation is used, hence alcohol recovery steps are not needed. Different grades of refined carrageenan and semi-refined carrageenan, such as Philippine natural grade (PNG) and processed *Eucheuma* seaweed (PES), have been developed to have a quality suitable for human consumption whilst being less expensive than mere refined carrageenan [96].

Agar is typically extracted by solubilization in hot water. For some species such as *Gelidium*, hot water is used directly after an initial cleaning step to extract agar, but for other species such as *Gracilaria*, alkali treatment is essential before hot water extraction, to improve the gel strength (Table 2b), otherwise the agar quality is too low for commercial applications. Similar to carrageenan, the extraction time, temperature and alkali concentration have to be optimized for individual seaweed species. Following alkali treatment, extensive rinsing is essential to neutralize residual alkali. Hot water extraction can also be combined with pressure to improve the extraction yield [43,86,97].

Alkali treatment in the extraction of agar and carrageenan does not always cause an improvement in the yield, but enhances the gel strength significantly, which can be observed in Table 3. During the washing step, after alkaline treatment, massive amounts of by-product are produced, which are currently poorly valorized. The study from Lebbar et al. [43] showed that the alkali waste contained low molecular weight compounds belonging to the glycerol-galactoside family, which have the potential for industrial applications.

### 5.3. Extraction of Green Seaweed Polysaccharides

The extraction of ulvan (the most well-known polysaccharide from green algae) has recently been reviewed by Kidgell et al. [27], and hence only a brief summary is included here. In accordance with the data found for other seaweed polysaccharide extractions (see Table 3), the quantitative yield was shown to vary significantly, ranging from 2.7–40% of the dry weight (expressed as median% of algal dry weight), and was dependent on both the applied process and the source of biomass.

The authors also highlighted that the main focus in published literature is on the physicochemical composition of the polymer and its interaction with the cell wall, while less attention is generally given to the risk of degradation and eventual co-extracted products. Factors of importance were identified to be pH, temperature and duration of extraction [27].

## 6. Co-Extraction of Seaweed Byproducts

The extraction of seaweed cell wall polysaccharides will, in most of the extraction processes, result in co-extraction of other polysaccharides (e.g., mixtures of alginate/fucoidans in brown seaweed or of ulvan/glucuronan in green seaweed) [22], proteins from cell wall polysaccharides-protein complex, and minerals which are adsorbed to the charged cell wall polysaccharides. These co-extracted byproducts make the extraction process challenging, requiring more purification steps to obtain the pure target polysaccharides. Sequential extraction of all seaweed compounds can be a solution to this, in order to extract the target compound selectively and also take advantage of the extraction of multiple compounds for different applications [98].

Many of the extraction processes described above result in products that in addition to the target polysaccharide also contain minerals (unpublished data). Brown macroalgae have a high content of ash, which mainly corresponds to different minerals, and have for example been reported as good sources of sodium (Na) and potassium (K), and can be used in the place of salt in food. They are also very good contributors of copper (Cu), zinc (Zn), molybdenum (Mo) and selenium (Se). Red and green macroalgae contain high amounts of manganese (Mn) and green algae are a great source of iron (Fe). Many macroalgae are also rich in iodine (I) [99]. An analysis of these minerals is thus important, especially if the products are to be considered for consumption, to take into account recommended daily allowances (RDAs) of minerals and trace elements [99,100]. Moreover, the absorption of heavy and toxic metals by algae (e.g., arsenic (As), cadmium (Cd) and lead (Pb)), has raised concerns about macroalgae consumption. Arsenic can occur in two forms: organic form (which has shown very low or no toxicity), or inorganic form (which contrarily has presented high toxicity), making analysis of the two forms important [101,102]. Generally, brown macroalgae accumulate As to a greater extent than red and green algae. High amounts of Cd and Pb have also been reported in the brown and red algae from some sites [99]. It can be concluded that the consumption of macroalgae should conform with the tolerable daily intake (TDI) established by the World Health Organization (WHO).

## 7. Modification of Seaweed Polysaccharides

### 7.1. Fucoidan

The depolymerization of crude fucoidan is considered necessary for effective treatment in biomedical applications; both due to the heterogeneity of fucose containing sulfated polysaccharides (FCSP) and due to the large molecular sizes. Fucoidan of low molecular weight has namely demonstrated better absorption and bioavailability compared with compounds of larger molecular weight [103]. The structural data of fucoidan suggest that there is no coherent basic structure of the polysaccharide, but rather a large variation between species, with varying compound sizes and sulfate groups substituted intermittently at C-2, C-3 and C-4. An example of fucoidan structure is presented in Table 1. Further investigation of the grade and position of sulfation in combination with compound size and linkage type is crucial for understanding the bioactivity of the compound [75].

Creation of oligosaccharides from sulfated polysaccharides is postulated as a way of controlling the compound size and its bioactivity. Enzymatic degradation by hydrolysis, using glycosidases such as endo- and exo-fucoidanases (also fucosidase, EC 3.2.1.44 and EC 3.2.1.51), can thus be valuable for the generation of oligosaccharides of lower molecular weight. Three types (suggested denomination ‘Type 1-3’ by Ale and Meyer [75]) of fucoidanases with different cleavage mechanisms are currently identified, all classified under glycoside hydrolase 107 in the CAZy database (GH107, see www.cazy.org). Type 1 catalyzes the endo-acting cleavage of α-1,4-glycosidic bonds, while Type 2 correspondingly catalyzes the cleavage of α-1,3-glycosidic bonds. Type 3, however, includes α-l-fucosidase, which are exo-acting enzymes that catalyze the degradation of α-l-fucosyl bonds at the non-reducing termini, releasing fucose from the backbone of fucoidan [75]. These enzymes occur in different GH-families in the CAZy database, of which the enzymes classified under GH29 are structurally related to the GH107 endofucoidanases [8].

Another important group of enzymes for the modification of fucoidan structure and bioactivity is sulfatases (EC 3.1.5.6). Modifying the degree of sulfation, by sulfation or de-sulfation, is important to control the effects of fucoidan and increase its biofunction [104]. Sulfatases are a uniform group of enzymes with highly conserved sequences, structures, and mechanisms. However, four different classes of enzyme are distinguished. Type I, formylglycine dependent sulfatase, comprises the majority of the identified sulfatases to date. Type II consists of Fe(II) α-ketoglutarate-dependent alkylsulfatases and Type III contains enzymes involved in Zn^2+^- or Mn^2+^-dependent metallo-β-lactamase activity. Type II and III do not encompass any sulfatases active in carbohydrates. Finally, Type IV is another potential sulfatase family member, with predicted marine polysaccharide activity [104].

The reported activity of sulfatases on polysaccharides and their specificity to oligosaccharides suggest potential in the effective modification of marine sulfated compounds. Most recognized sulfatases are constructed of one catalytic module, but dimodular and multimodular enzymes also occur. Structural analyses indicate that sulfatases with human and bacterial origins have a similar structure [104,105]. They act by catalyzing the hydrolytic desulfonation mechanism of sulfate esters (CO-S) and sulfamates (CN-S).

### 7.2. Alginate

Within the pharmaceutical industry, alginic acid and alginates are primarily used as excipients in formulations for oral and topical administration, due to their physiochemical properties. The primary usage is for the assurance of controlled drug release, which is affected by systemic pH and temperature, as well as the binding strength of alginate. G-residues of alginates have a stronger affinity to divalent ions compared with M-residues, as they crosslink in the form of a pocket (denominated ‘the egg-box’) surrounding the ion, generating alginates with significantly higher strength. Therefore, the physiochemical properties are highly dependent on the M/G ratio of the polymer. The properties of alginate products can accordingly be altered by enzymatically modifying the M/G ratio [106,107,108].

Four groups of alginate modifying enzymes have been characterized: mannuronan C-5-epimerases, alginate lyases, alginate acetylases, and alginate deacetylases [106]. Mannuronan C-5-epimerases are involved in the epimerization of mannuronic acid to guluronic acid, increasing the crosslinking properties of alginate. Alginate lyases are well studied and have been demonstrated to catalyze alginate degradation with different affinities to the four different bonds (M–M, G–G, M–G, G–M) of the polymer. Most alginate lyases operate by endolytic modes, generating oligomers usually in the size range of two to five monomers. However, some alginate lyases are not able to accommodate acetylated substrates in their active site. Therefore, to facilitate or hinder enzymatic hydrolysis, alginate acetylases and alginate deacetylases, respectively, can be utilized to modify the accessibility of the enzyme to the substrate. Moreover, the degree of acetylation affects the water-binding properties and increases the viscosity of the alginate.

### 7.3. Laminarin

Complex and highly potent structures could be engineered from laminarin as health-promoting agents, if appropriate enzymes are accessed and used. Hydrolytic enzymes, including endo-β-1,3-glucanases and -d-glucosidases (EC 3.2.1.6 and EC 3.2.1.39), as well as exo-β-1,3-glucanases (EC 3.2.1.58), can be used for depolymerization reactions of the laminarin backbone structure, to generate possibly more potent bioactive derivatives. Endo-acting enzymes act randomly by catalyzing the hydrolysis reaction of the backbone structure into glucose and oligosaccharides, while exo-enzymes catalyze hydrolysis of the reducing end into glucose. Although β-1,3-glucanases are a well-studied group of enzymes, their β-1,6-linked side-chain analogues are less explored. Endo-acting enzymes can be limited by steric hindrance from side groups in highly branched laminarins, resulting in reduced enzyme activity [109].

Of perhaps greatest interest is the use of laminarin oligosaccharides as anti-inflammatory and anticancer agents, relating to the potential effect of β-1,3-(1,6)-glucans. They bind primarily to the receptor Dectin-1 [110], a type II transmembrane receptor expressed in high levels on blood and splenic monocytes, neutrophils and alveolar and inflammatory macrophages, and at lower levels on dendritic cells [110,111]. These properties are neither found in mixed β-1,3- and β-1,4-linked glucans, 1,6-glucans (pustulan), nor in 1,4-glucans [37]. The determinants of these important activities are, however, not completely resolved, but they appear to relate to the size and content of β-1,6-linkages as well as their type (branch points or kinks). β-1,3-linked oligosaccharides smaller than heptose do not bind to Dectin-1. The smallest oligosaccharide binding to recombinant Dectin-1 is a β-1,3-d-glucan oligomer, having a degree of polymerization of 7 (DP 7), with a single β-1,6-linked glucose unit [37]. Laminarin-derived oligosaccharides have also been shown to inhibit the proliferation of U937 leukemia cells to a greater degree than the source laminarins from *L. digitata* and *E. bicyclis* [112]. A NMR analysis indicated that the active β-glucan oligomers were larger than DP 8 with a ratio of 3:1 between β-1,3- and β-1,6-linkages. Oligosaccharides with a higher degree of branching derived from *E. bicyclis* laminarin elicited a stronger reaction. Hreggviðsson et al. [113,114,115] have developed efficient transglucosidases, which are highly promising as glucan engineering tools for making potent bioactive laminarin oligosaccharides, as they can catalyze, depending on the enzyme, β-1,3-elongation, β-1,6-branching or β-1,6-interchain kinking of β-1,3-glucan oligomers.

### 7.4. Carrageenan

There are many studies on enzymes hydrolyzing plant polysaccharides, but enzymes degrading marine polysaccharides are not largely explored [116]. Indeed, enzymes acting on marine polysaccharides must be adopted to accommodate the sulfate group of marine polysaccharides which is not available in plant polysaccharides. Carrageenan is the most well-known sulfated marine polysaccharide from red seaweed. Enzymes acting on carrageenan belong to different glycoside hydrolase (GH) families listed in in the carbohydrate-active enzymes (CAZy) database. The enzymes acting on κ-carrageenan (κ-carrageenase) and ι-carrageenan (ι-carrageenase) belong to the families GH16 and GH82, respectively [117,118], while there is no specified GH family for λ-carrageenase.

κ-carrageenases are endo-acting enzymes that hydrolyze the β-1,4-linkage between d-galactose-4-sulfate and 3,6-anhydro-d-galactose residues. The endo-hydrolase ι-carrageenase cleaves the β-1,4-linkages between the d-galactose-4-sulfate and 3,6-anhydro-d-galactose-2-sulfate in ι-carrageenan. λ-carrageenase is also an endo-acting enzyme hydrolyzing the glycoside linkage in λ-carrageenan backbone-producing tetrasaccharides [119].

### 7.5. Agar

Agarases are enzymes that catalyze the hydrolysis of agarose. They can be intra-cellular or extra-cellular and are isolated from different genera of bacteria available in seawater and marine sediments, marine algae, marine mollusks, fresh water, and soil. Two types of agarase are classified according to their cleavage pattern; α-agarase (E.C. 3.2.1.158) and β-agarase (E.C. 3.2.1.81) [120,121]. α-agarase hydrolyzes the α-1,3-linkages in agar backbone producing agarooligosaccharides (AOs) with 3,6-anhydro-l-galactopyranose as the reducing end [122], while β-agarase cleaves the β-1,4-linkages producing neoagarooligosaccharides (NAOs) with d-galactose as the reducing end [120].

In addition to the production of oligosaccharides from agar with potential application in pharmaceutical industries, agarases are also used for the recovery of DNA from agarose gel and preparation of protoplasts of the seaweed to recover substances with biological activities, including unsaturated fatty acids, vitamins, and carotenoids [120].

### 7.6. Ulvan

The enzymatic depolymerization mechanisms of ulvan, the major polysaccharide in green seaweed, remain largely unknown [123]. Ulvan is degraded by ulvan lyases by β-elimination mechanisms releasing oligosaccharides with unsaturated uronic acids at the nonreducing end. Some of the characterized ulvan lyases are classified into the polysaccharide lyase family 24 (PL24), PL25 or PL28 in the CAZy database. To date, only a few ulvan lyases from marine bacteria have been isolated and characterized, but have not been industrially employed further. Research on the isolation and characterization of ulvan lyases is ongoing, but more investigation is needed to recognize the usability of these enzymes. Additionally, the applicability of hydrolyzed products in the production of value-added chemicals is investigated.

## 8. Current and Potential Novel Applications

Many coastal countries, especially Japan, Korea and China, have the culture of using macroalgae in their food as ingredients in different soups, combined with fish or meat, or as a substitution for vegetables. Examples of some edible macroalgae include *Ulva* spp., *Enteromorpha* spp., *Caulerpa* spp., *Codium* spp., *Monostroma* spp., *Sargassum* spp., *Hydroclathrus* spp., *Laminaria* spp., *Undaria* spp., *Macrocystis* spp., *Porphyra* spp., *Gracilaria* spp., *Eucheuma* spp., *Laurencia* spp., and *Acanthophora* spp. [124].

Red algae have historically been consumed as feed [125], food, food ingredients and food supplements. For example, *Palmaria palmata*, also called dulse or dillisk, which grows on the northern coast lines of Atlantic and Pacific oceans, has been a part of the human diet in Iceland, Ireland and Scotland for a long time, and its popularity is increasing in other western countries [126,127]. *Porphyra* species including *Porphyra tenera, Porphyra pseudolinearis* and *Porphyra yezoensis*, also known as Nori, are other well-known species grown mainly in Japan, the Republic of Korea, and China. These species are reported as being among the most nutritious types of algae. They have a long history in the human diet in many Asian countries and the consumption of these species has shown an increasing trend in other countries as well [128,129]. In addition, red algae can have potential as a meat substitute due to its relatively high protein content and amino acid composition [130].

In vitro studies of bioactive compounds in macroalgae have shown health promoting effects on the human body. However, more in vivo trials are required in order to support the claims of the health promoting effects of macroalgae. Additionally, sustainable and cost-effective pretreatment, extraction and purification methods are needed with zero waste for industrial application [131]. Nutritional compatibility, low production cost, constant accessibility, and enough quality are the main prerequisites for commercialization [132]. Although seaweed as a whole product is emerging globally, extracted polysaccharides from algae have received much research attention. This review focuses on the most prominent polysaccharides in brown, red and green seaweeds.

### 8.1. Fucoidan

Fucoidan has been of interest in many applications due to its potential biomedical effects, including anticarcinogenic, antimicrobial, anticoagulant, antiviral, immunomodulatory, and antioxidant effects [133]. However, although the biomedical potential of fucoidan has been studied comprehensively in vitro, and to some extent in vivo in non-human animal systems, only a few clinical studies on humans have been performed. Studies on orally administrated fucoidan have indicated that they are urinarily excreted. However, further studies on the absorption, distribution, metabolism and excretion of fucoidan are required to understand its systemic effects [134]. A phase I clinical study (ClinicalTrials.cov, NCT03422055) on tolerance, biodistribution and dosimetry of radio-labelled fucoidan on healthy volunteers is currently ongoing. The study primarily aims to investigate the potential adverse effects and serious adverse events after intravenous administration of the drug.

The anticarcinogenic properties of fucoidan have probably received the most attention so far and have been studied in several in vitro systems to assess the mechanisms of action towards various types of cancer. Several papers review the potential applications of fucoidan in cancer treatment, and these also present which anti-cancer mechanisms have been studied to date [103,135,136]. Most studies presented in the literature are performed in vitro or in vivo in non-human animal expression systems. However, a phase II clinical study (ClinicalTrials.gov, NCT04066660), primarily investigating the disease control rate of oligo-fucoidan as a dietary supplement for patients with advanced hepatocellular carcinoma, is currently recruiting.

Although clinical evidence so far on the effectivity and bioavailability of fucoidan is scarce, several dietary supplements are marketed as capsules, liquids and powders, with claimed indications such as support of a healthy immune system, optimal cellular aging, a healthy digestive function, and a healthy inflammation response.

### 8.2. Alginate

Alginate is currently used in a wide range of industries for applications such as feed stabilizer, paper and welding rod coatings, and dye thickener in textile printing [77]. The polysaccharide is also widely used in the food and drink industries due to its gelling, thickening, stabilizing and emulsifying properties. The salts of alginic acid with monovalent cations form a viscose solution in water. The viscosity of alginate solutions is affected by alginate concentration and molecular weight, pH [137], and temperature [138]. Sodium alginate is not effective in acidic solutions because insoluble alginic acid is formed. In mild acidic conditions, propylene glycol alginate (PGA) is preferred, since it demonstrates a higher grade of stability. PGA, an emulsifier, stabilizer, and thickening agent, is an ester form of alginate in which some of the carboxyl groups are esterified with propylene glycol, some are neutralized with alkali, and some remain free. Alginate can form a hydrogel in the presence of divalent cations due to the crosslinking of the carboxylate groups of G blocks in the polymer backbone, constituting the egg-box model. This ability makes alginate a potential candidate for the production of edible films and coatings for food packaging [139]. More information on the applications of alginate as a food additive was published in a comprehensive review by Qin et al. [140]

In addition to its use in the chemical, food and feed industries, alginates are also used in pharmaceutical applications. Although alginates are already utilized as excipients in drug delivery systems, they are of interest as primary ingredients in some pharmaceutical applications. Several clinical studies on human subjects focus on alginate applications for symptoms such as; obesity, diabetes, dry eyes, gastroesophageal reflux, cystic fibrosis and topical disorders (clinicaltrials.gov, search term ‘alginate’). In particular, studies linking alginate to decreased small intestinal fat absorption and increased satiety have been conducted and have generated varying results [141,142,143].

### 8.3. Laminarin

Commercial applications of laminarin are limited, and so far the amount of research is inferior to other polysaccharides of brown algae. However, similar to other β-1,3-(1,6)-glucans, laminarin is reported to have a broad range of biological activities and to have similar potential for applications in functional foods, nutraceuticals, pharmaceuticals, and cosmeceuticals [144]. Laminarin from brown seaweed is used in agricultural applications where it has been shown to limit pathogen infections by stimulating plant defense systems [145], and is commercialized as Vacciplant^®^ [146]. Additionally, unprocessed marine macroalgae have historically been used as agricultural nourishment and are today commercialized as organic liquid fertilizer.

Since laminarin does not possess any chelating properties, its primary potential emerges within medical and nutraceutical applications. It is suggested that laminarin has potential applicability as a dietary fiber [33,147]. Dietary fibers, divided into soluble and insoluble fibers, are carbohydrates that cannot be degraded by the endogenous enzymes of the body and are either fermented in the colon or provide bulking. Laminarin from *S. latissima* has been demonstrated to evade hydrolysis in the upper intestinal tract, while not being excreted with the feces of rat models, suggesting fermentation in the colon. Laminarin has been shown to exert beneficial metabolic effects on gut health as a modulator of intestinal metabolism through its effects on mucus composition, intestinal pH, and short-chain fatty acid (SCFA) production, especially butyrate, by the gut microbiota [148]. However, although studies suggest the prebiotic effects of laminarin, further studies on the digestibility, bioavailability and biodistribution are required to better understand the systemic effects of the compound.

Laminarin may also be used as an immunomodulating and anti-cancer agent. These effects are observed in related glucans and can be attributed to specific glycosidic β-1,3-(1,6)-linkage patterns [149,150,151] that modulate the immune response [152] and inhibit cell proliferation in HT-29 colon cancer cells. On the basis of such properties, derivatives of insoluble β-1,3-(1,6)-glucans from yeast are currently being developed as ‘imprime PGG’ by the company Biothera as intravenous agents against cancer, affecting the innate immune system. Dietary products (Yestimun) developed from unmodified yeast β-1,3-(1,6)-glucan and based on its immunomodulatory effects [153] are also commercialized.

### 8.4. Carrageenan

Carrageenan is used in a wide variety of applications in the food industry; mainly in dairy and meat applications, based on its physiochemical properties as a thickening, gelling, and stabilizing agent. Various types of carrageenan can show a broad range of gelling and emulsifying properties, such as rigidity or elasticity, transparency or turbidity, toughness or tenderness, heat stability or thermal reversibility, as well as low or high melting/gelling temperatures. These physiochemical properties are affected by the chemical structure of carrageenan, the type and concentration of cations including salts and proteins present in the solution, and the heating and cooling profile of the carrageenan solution. In fact, ι-carrageenan can form an elastic gel in the presence of calcium salt, while κ-carrageenan forms a brittle gel in the presence of calcium but forms a strong and rigid gel with potassium. On the other hand, λ-carrageenan is not gel-forming in the presence of divalent salts, but develops a highly viscous solution [154,155]. However, it exhibits gelation in the presence of trivalent ions [41].

Carrageenan has potential applications in the pharmaceutical industry because of its biological activities including anticoagulant [156], antiviral [157,158], cholesterol-lowering [159], anti-tumor and immunomodulatory [160,161,162], and antioxidant activities [163,164]. Degradation of carrageenan polysaccharides into smaller compound structures could be a way of controlling the bioactivity. Although oligosaccharides derived from carrageenan have reported antitumor [165], antioxidant activity [166] and immune regulation activities [167], which make them very valuable in the pharmaceutical industry, carrageenan also has various applications in bioethanol production, in the textile industry, as a detergent additive and in the isolation of the protoplast of algae [168].

Recent applications of carrageenan in the pharmaceutical industry were summarized by Li et al. [169], including as a polymer matrix in oral extended-release tablets, as a novel extrusion aid for the production of pellets, and as a carrier/stabilizer in microparticle/nanoparticle systems. Moreover, carrageenan is used in the production of nanoengineered injectable hydrogels in tissue regeneration therapy [170]. Other potential applications of carrageenan include utilization as fertilizer, and studies by Vera et al. [171] showed that oligo-carrageenan induces a broad range of protection against pathogens in plants.

### 8.5. Agar

Agar is widely used as a gelling agent due to its unique physiochemical properties which enable gel formation in water at temperatures around 38 °C and facilitate melting at temperatures around 85 °C [172]. In addition to food application, including bakery, confectionery, dairy products, canned meat and fish products [173], agar is used in the preparation of bacteriological culture media [174]. Agarose, a fraction of agar with a lowest possible charge, is used for electrophoretic separation in agarose gel electrophoresis [175], the preparation of porous beads for chromatographic protein purifications, and mobility assay [176,177]. Agarose is also used as a support for the three-dimensional cultures of human and animal cells [178,179].

### 8.6. Ulvan

Sulfated polysaccharides from marine seaweeds have recently received attention in the food, cosmetics and pharmacology areas. Sulfated polysaccharides can originate from red (carrageenans) or brown (fucoidans) macroalgae, but are also present in the form of ulvans in green seaweeds.

Ulvan has been reported to mimic glycosaminoglycans in animals, and hence mimic these types of activities. For this purpose, sulfation is proposed as important [27], with the degree of sulfation and sulfation patterns influencing the bioactivity. In addition, the molecular weight, constituent sugars linkages and degree of branching are suggested as factors that influence bioactivity. The bioactivities reported from green seaweeds, include antioxidant activity, shown from *Ulva pertusa* [180], immunomodulating activity (from sulfated polysaccharides of e.g., *Monostroma nitidum* and *U. pertusa* [181,182], antihyperlipidemic activity (reported from *U. pertusa*) [183], anticoagulant activity from *M. nitidum* and anticancer from, for example, *M. nitidum* [181,184].

Recently, ulvans have also been found to influence plant immunity, including stimulation of the inducible defenses involving recognition of microbes/pathogens. In this field, the sugar constituents are reported as important, and rhamnose has for instance been shown to be important for triggering plant defense in several different plants, including apple leaves, tomatoes, and thale cress (*Arabidopsis thaliana*), as reviewed by Kidgell et al. [27].

## 9. Conclusions and Future Aspects

Although polysaccharides from marine macroalgae have comprehensively reported potential in valuable biomedical applications, commercial products are scarce on the market. Many of the numerous bioactivities reported are still only at research level, and more work remains before reaching mature application. Today, marine macroalgal polysaccharides are mainly used industrially for their distinctive physiochemical properties as additives or excipients. It is obvious that the extraction processes of polysaccharides are crucial for the properties of the extractive. Strong acids and bases can exert uncontrolled chemical degradation and introduce unwanted structural changes that alter the bioactive properties of the polysaccharides. Yet, novel extraction methods are not optimized for an industrial scale. Additionally, extraction yields are sometimes remarkably low, and require improvements for industrial realization.

Polysaccharides exert a large structural variation depending on factors such as algae species, harvest season, and growth geography, as well as extraction and purification methods, which is problematic for commercialization. A few products from macroalgal polysaccharides are currently marketed, although clinical evidence is scarce. One of the bottlenecks in commercializing the bioactivities lies in the complex structure of the glycans, and enzymatic deconstruction into more defined small units may be a way of achieving better defined molecules which are more suitable for the applications. Enzymatic treatment, as mentioned, can be a useful tool for necessary modification of the compound structure to achieve structures with bioactivity in humans. However, better knowledge about structures and activities of both enzymes and bio-compounds are crucial.

## Figures and Tables

**Table 1:** Basic structures of polysaccharides derived from marine macroalgae: (a) alginate, (b)–(c) fucoidan, (d) laminarin, (e)–(f) carrageenan, (g)–(h) agar, and (i)–(k) ulvan (where (i)–(j): ulvanobiuronic acids and k: ulvanobiose).

Polysaccharide	Structure ^1^	
Alginate	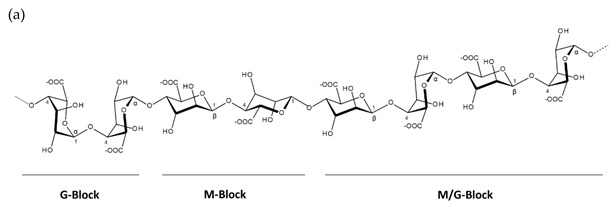 β-1,4-d-mannuronic acid (M) and α-1,4-l-guluronic acid (G) residues forming GG, MM and M/G blocks	
Fucoidan	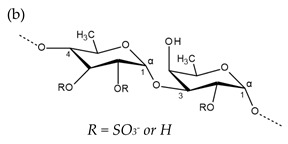 Alternating 1,3- and 1,4-linked α- l-fucopyranose	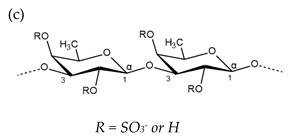 α-1,3-l-fucopyranose
Laminarin	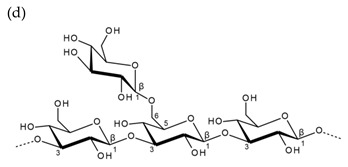 β-1,3-d-glucopyranose backbone with branching β-1,6-d-glucopyranose unit	
Carrageenan	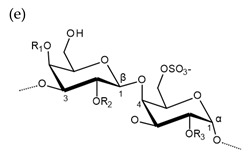 µ-carrageenan: R_1_ = SO_3_^−^, R_2_ = R_3_ = H ν-carrageenan: R_1_ = R_3_ = SO_3_^−^, R_2_ = H λ-carrageenan: R_1_ = H, R_2_ = R_3_ = SO_3_*^−^* Alternating α-1,4-d-galactopyranose and β-1,3-d-galactopyranose	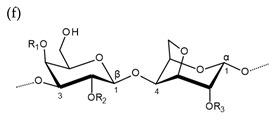 κ-carrageenan: R_1_ = SO_3_^−^, R_2_ = R_3_ = H ι-carrageenan: R_1_ = R_3_ = SO_3_^−^, R_2_ = H θ-carrageenan: R_1_ = H, R_2_ = R_3_ = SO_3_ Alternating β-1,3-d-galactopyranose and 3,6-anhydro-α-d-galactopyranose
Agar	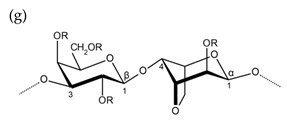	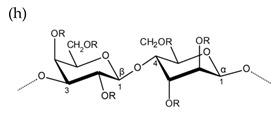
	R = H or side chain substituents e.g., sulfate ester, methoxyl ether or pyruvic acid	
	Alternating β-1,3-d-galactopyranose and 3,6-anhydro-α-1,4-l-galactopyranose	Alternating β-1,3-d-galactopyranose and α-1,4-l-galactopyranose
Ulvan	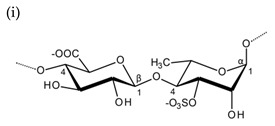 Alternating β-1,4-d-glucuronic acid and α-1,4-l-rhamnopyranose (from *Ulva rigida*)	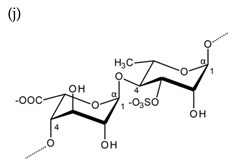 Alternating α-1,4-l-iduronic acid and α-1,4-l-rhamnopyranose (from *Ulva armoricana*)
	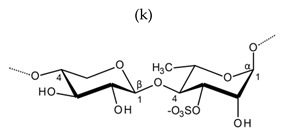 Alternating β-1,4-d-xylanopyranose and α-1,4-l-rhamnopyranose (as shown in *Enteromorpha* sp)	

^1^ Structures were drawn using BIOVIA Draw, based on structures from Synytsya et al. [22], Zhao et al. [23], Campo et al. [24], Tuvikene et al. [25], Pérez et al. [26], and Kidgell et al. [27]

**Table 2:** Overview of extraction methods.

Extraction Method	Principle	Advantages	Disadvantages	Ref.
**Conventional chemical extraction**	Different procedures of acid or alkaline extraction and hot or cold water extraction. Acidic or alkaline conditions are usually applied to facilitate extraction, as hydrogen ions (H^+^) and hydroxyl ions (OH^−^) interfere with hydrogen linkages between polysaccharides. The conventional extraction procedures rely on the solubility properties of target compounds and are often preceded by pretreatment steps where lipids, pigments, proteins and other impurities are removed by solvents.	Well established methods.	Long extraction time. High consumption of energy and water. Alcohol precipitation and recovery are costly. Chemical solvents may have health hazards. Acids and bases can cause degradation of the target polysaccharide to compounds of smaller molecular size.	[56,57,58,59,60,61]
**Ultrasound assisted extraction (UAE)**	UAE acts by exposing the biomass to sound waves of high frequencies larger than 20 kHz. Strong ultrasound fields cause the implosion of vapor bubbles in liquids formed under high pressure conditions. Implosion of these bubbles in near proximity to liquid-solid borders, such as cell walls, subject these solid surfaces to strong forces resulting in cell breakdown. UAE can be performed at low temperatures which enable the extraction of thermosensitive target compounds.	Short extraction time. Higher yields of extracted bioactives. Simple method to operate. Operation at low temperatures. Relatively low amounts of solvent are required.	Degradation and structural changes in the structure of polysaccharides.	[54,62,63]
**Microwave assisted extraction (MAE)**	MAE utilizes non-ionizing electromagnetic radiation with frequencies between 300 MHz and 300 GHz which cause the disruption of hydrogen bonds and migration of dissolved ions. This enables the solvent to enter the cell matrix and facilitate the withdrawal of compounds of interest. Variables such as temperature, pressure, time and algae/water ratio can be altered to optimize the yield of the desired product.	Short extraction time. Relatively low amounts of solvent are required. Higher quality of product.	Lower yield is achieved due to degradation.	[54,61,64,65]
**Enzyme assisted extraction (EAE)**	EAE operates by using enzymes for degradation of the algal cell wall, thereby releasing target compounds. Critical parameters, including pH, temperature and treatment time, should be optimized for specific enzymes to maximize the extraction result. Enzymes catalyzing degradative reactions of cell wall structure compounds like cellulose, β-glucan and hemicellulose are usually used to facilitate the extraction of target molecules.	Relatively low amounts of solvent are required. Relatively low-cost technique. Disruption of cell wall components is enzymatically performed. Original efficacy of bioactives is preserved to a high degree. Potential of a higher yield of the target compound. The mild conditions applied to the sample during EAE are advantageous when isolating sensitive bioactive compounds.	Extraction yield is dependent on optimum treatment time, pH and temperature conditions of enzymes. Target compounds risk being degraded by non-specific enzymes. Extraction efficiency is dependent on enzyme properties.	[54,66,67,68,69]
**Supercritical fluid extraction (SFE)**	A supercritical fluid is a substance at a temperature and pressure above its critical point, where gas and liquid phases are indistinct. Altering the two parameters can change the solubility of the fluid. Carbon dioxide is commonly used as its critical point is relatively low and thus requires less energy input to become supercritical, compared to substances with higher critical points. Supercritical fluids are characterized for their low viscosity and high diffusivity which gives them better transport properties than liquids. SFE is considered an environmentally friendly process as it does not require the use of solvents. However, procurement costs are high in relation to other extraction methods. This concept is therefore predominantly employed to extract highly valuable compounds.	Use of non-toxic and non-flammable solvent. Supercritical CO_2_ is inexplosive, readily available, and can be removed easily from the final extract. Does not cause degradation and structural disruptions in bioactive compounds.	High pressure is needed to maintain the solvent in critical state, which can have negative effect on compounds. Relatively high procurement costs.	[54,70,71,72]

**Table 3:** Extraction yields from different extraction methods.

Polysaccharide	Yield ^1^	Species	Method	Ref.
**Fucoidan**	1.63%	*Padina tetrastromatica*	Conventional extraction (HCl)	[74]
	9.46%	*P. tetrastromatica*	Conventional extraction (hot water)	[74]
	3.51%	*Nizamuddinia zanardinii*	UAE	[80]
	4.44% (4.7%, CaCl_2_)	*Fucus evanescens*	UAE	[81]
	18.22%	*Fucus vesiculosus*	MAE	[64]
	16.08% (20.98%, HCl)	*Ascophyllum nodosum*	MAE	[65]
	5.58%, Alcalase 4.80%, Cellulase 4.36%, Flavourzyme 4.28%, Viscozyme (5.20%, hot water)	*N. zanardinii*	EAE	[82]
	1.5 ± 0.3%	*U. pinnatifida*	EAE	[83]
	3.02% (5.11%, EtOH)	*F. evanescens*	SFE	[84]
	1.26% (1.35%, SFE-EtOH) (1.28%, EtOH)	*S. japonica*	SFE	[84]
	0.57% (0.55%, SFE-EtOH) (0.65%, EtOH)	*Sargassum oligocystum*	SFE	[84]
**Alginate**	51.8%	*L. digitata*	Conventional extraction (HCl)	[79]
	13.47%	*Sargassum muticum*	Conventional extraction (HCl)	[78]
	54% ^2^	*Sargassum binderi*	UAE	[85]
	23.6 ± 1.2%	*U. pinnatifida*	EAE	[83]
**Laminarin**	6.0 ± 0.7%	*S. latissima*	Conventional extraction (HCl + EtOH)	[33]
	19 ± 2.6%	*S. latissima*	Conventional extraction (HCl + NaOH)	[33]
	20%	*S. latissima*	Conventional extraction (hot H_2_SO_4_)	[33]
	6.1 ± 1.6%	*S. latissima*	Conventional extraction (hot HCl)	[33]
	3.2 ± 0.9%	*U. pinnatifida*	EAE	[83]
**Agar**	29.7 ± 1.9% Gel strength: 271 ± 38 g/cm^2^	*Gracilaria lemaneiformis*	Conventional extraction (hot water)	[86]
	25.8 ± 2.9% (Gel strength: 1761 ± 35 g/cm^2^)	*G. lemaneiformis*	Conventional extraction (alkali pre-treatment with 5% NaOH + hot water)	[86]
	25.4 ± 1.7% (Gel strength: 1913 ± 38 g/cm^2^)	*G. lemaneiformis*	Conventional extraction (alkali pre-treatment with 5% NaOH +Photobleaching + hot water)	[86]
	29.7–34.6% (Gel strength: 72 g/cm^2^)	*Gracilaria vermiculophylla*	Conventional extraction (hot water)	[87]
	15.03% (Gel strength: 1064 g/cm^2^)	*G. vermiculophylla*	Conventional extraction (pre-treatment with 7% NaOH + hot water)	[87]
**Carrageenan**	46.43%	*E. cottonii*	Conventional extraction (hot water)	[88]
	37.02%	*E. cottonii*	Conventional Extraction (KOH)	[88]
	76.3%	*Furcellaria lumbricalis Coccotylus truncates*	Conventional extraction (hot water)	[89]
	72.6%	*F. lumbricalis* *C. truncatus*	Conventional extraction (0.15 M NaOH)	[89]
	55.3%	*F. lumbricalis* *C. truncatus*	Conventional extraction (0.15 M KOH)	[89]
	50–55% (for both species) ^3^	*K. alvarezii* *Euchema denticulatum*	UAE	[85]
	28.65%	*Mastocarpus stellatus*	EAE	[90]

^1^ Based on dried biomass. ^2^ Ultrasound extraction resulted in doubling the extraction yield from 28% to 54%. ^3^ Longer ultrasound treatment of up to 30 min did not further improve the extraction yield. Additionally, in this study, degradation and structural modification were not observed in extracted polysaccharide.

## References

[1] Chapman V.J., Chapman D.J. (1980). Seaweeds and their Uses.

[2] FAO (2019). FAO yearbook. Fishery and Aquaculture Statistics 2017.

[3] Campbell I., Macleod A., Sahlmann C., Neves L., Funderud J., Øverland M., Hughes A.D., Stanley M. (2019). The Environmental Risks Associated With the Development of Seaweed Farming in Europe—Prioritizing Key Knowledge Gaps. Front. Mar. Sci..

[4] FAO (2018). The Global Status of Seaweed Production, Trade and Utilization.

[5] Jung K.A., Lim S.-R., Kim Y., Park J.M. (2013). Potentials of macroalgae as feedstocks for biorefinery. Bioresour. Technol..

[6] Field C.B., Behrenfeld M.J., Randerson J.T., Falkowski P. (1998). Primary Production of the Biosphere: Integrating Terrestrial and Oceanic Components. Science.

[7] Harrysson H., Hayes M., Eimer F., Carlsson N.-G., Toth G., Undeland I. (2018). Production of protein extracts from Swedish red, green and brown seaweeds, *Porphyra umbilicalis* Kützing, *Ulva lactuca* Linneus, and *Saccharina latissima* (Linneus), J.V. Lamoroux, using three different methods. J. Appl. Phycol..

[8] Hreggviðsson G.O., Nordberg-Karlsson E.M., Tøndervik A., Aachmanne F.L., Dobruchowska J.M., Linares-Pastén J., Daugbjerg-Christensen M., Moneart A., Kristjansdottir T., Slettad H., Torres M.D., Kraan S., Dominguez H. (2020). Biocatalytic refining of polysaccharides from brown seaweeds. Sustainable Seaweed Technologies—Cultivation, Biorefinery, and Applications.

[9] Milledge J.J., Harvey P.J., Rampelotto P.H., Trincone A. (2018). Anaerobic Digestion and Gasification of Seaweed. In Grand Challenges in Marine Biotechnology.

[10] Vijayaraghavan M.R., Sokhi G. (1986). Phaeophyceae—An Ultrastructural and Histochemical Overview. Proc. Indian. Natn. Sci. Acad..

[11] Mautner H.G. (1954). The Chemistry of Brown Algae. Econ. Bot..

[12] Deniaud-Bouët E., Kervarec N., Michel G., Tonon T., Kloareg B., Hervé C. (2014). Chemical and enzymatic fractionation of cell walls from Fucales: Insights into the structure of the extracellular matrix of brown algae. Ann. Bot..

[13] Deniaud-Bouët E., Hardouin K., Potin P., Kloareg B., Hervé C. (2017). A review about brown algal cell walls and fucose-containing sulfated polysaccharides: Cell wall context, biomedical properties and key research challenges. Carbohyd. Polym..

[14] Graiff A., Ruth W., Kragl U., Karsten U. (2016). Chemical characterization and quantification of the brown algal storage compound laminarin - A new methodological approach. J. Appl. Phycol..

[15] Kloareg B., Quatrano R.S. (1988). Structure of the cell walls of marine algae and ecophysiological functions of the matrix polysaccharides. Oceanogr. Mar. Biol. Annu. Rev..

[16] Salmeán A.A., Duffieux D., Harholt J., Qin F., Michel G., Czjzek M., Hervé C. (2017). Insoluble (1→3), (1→4)-β-D-glucan is a component of cell walls in brown algae (Phaeophyceae) and is masked by alginates in tissues. Sci. Rep..

[17] Lim S.J., Mustapha W.A.W., Maskat M.Y., Latip J., Badri K.H., Hassan O. (2016). Chemical Properties and Toxicology Studies of Fucoidan Extracted from Malaysian *Sargassum binderi*. Food Sci. Biotechnol..

[18] Ale M.T., Mikkelsen J.D., Meyer A.S. (2011). Important Determinants for Fucoidan Bioactivity: A Critical Review of Structure-Function Relations and Extraction Methods for Fucose-Containing Sulfated Polysaccharides from Brown Seaweeds. Mar. Drugs.

[19] Jiao G., Yu G., Zhang J., Ewart H.S. (2011). Chemical Structures and Bioactivities of Sulfated Polysaccharides from Marine Algae. Mar. Drugs.

[20] Fletcher H.R., Biller P., Ross A.B., Adams J.M.M. (2017). The seasonal variation of fucoidan within three species of brown macroalgae. Algal Res..

[21] Starko S., Mansfield S.D., Martone T. (2018). Cell wall chemistry and tissue structure underlie shifts in material properties of a perennial kelp. Eur. J. Phycol..

[22] Synytsya A., Čopíková J., Kim W.J., Park Y.I., Kim S.-K. (2015). Cell Wall Polysaccharides of Marine Algae. Springer Handbook of Marine Biotechnology.

[23] Zhao X., Jiao G., Wu J., Zhang J., Yu G., Liu Y., Wang Z., Zhang J. (2015). Laminaria japonica Aresch. and Ecklonia Kurome Okam. 昆布 (Kunbu, Kelp). Dietary Chinese Herbs.

[24] Campo V.L., Kawano D.F., da Silva D.B., Carvalho I. (2009). Carrageenans: Biological properties, chemical modifications and structural analysis—A review. Carbohyd. Polym..

[25] Tuvikene R., Truus K., Robal M., Volobujeva O., Mellikov E., Pehk T., Kollist A., Kailas T., Vaher M. (2010). The extraction, structure, and gelling properties of hybrid galactan from the red alga *Furcellaria lumbricalis* (Baltic Sea, Estonia). J. Appl. Phycol..

[26] Pérez M.J., Falqué E., Domínguez H. (2016). Antimicrobial Action of Compounds from Marine Seaweed. Mar. Drugs.

[27] Kidgell J.T., Magnusson M., deNys R., Glasson R.K. (2019). Ulvan: A systematic review on extraction, composition and function. Algal Res..

[28] Zhao X., Li B., Xue C., Sun L. (2012). Effect of molecular weight on the antioxidant property of low molecular weight alginate from *Laminaria japonica*. J. Appl. Phycol..

[29] Brownlee I.A., Seal C.J., Wilcox M., Dettmar P.W., Pearson J.P., Rehm B.H.A. (2009). Applications of Alginates in Food. Alginates: Biology and Applications.

[30] Miller I.J. (1996). Alginate Composition of Some New Zealand Brown Seaweeds. Phytochemistry.

[31] Skjåk-Bræk G., Donati I., Paoletti S., Matricardi P., Alhaique F., Coviello T. (2015). Alginate hydrogels: Properties and applications. Polysaccharide Hydrogels: Characterization and Biomedical Applications.

[32] Domozych D. (2019). Algal Cell Wall. Encyclopedia of Life Sciences.

[33] Devillé C., Damas J., Forget P., Dandrifosse G., Peulen O. (2004). Laminarin in the dietary fibre concept. J. Sci. Food Agric..

[34] Kadam S.U., Tiwari B.K., O’Donnell C.P. (2014). Extraction, structure and biofunctional activities of laminarin from brown algae. Int. J. Food Sci. Tech..

[35] Read S.M., Currie G., Bacic A. (1996). Analysis of the structural heterogeneity of laminarin by electrospray-ionisation-mass spectrometry. Carbohydr. Res..

[36] Maeda M., Nisizawa K. (1968). Fine structure of laminaran of *Eisenia bicyclis*. J. Biochem..

[37] Adams E.L., Rice P.J., Graves B., Ensley H.E., Yu H., Brown G.D., Gordon S., Monteiro M.A., Papp-Szabo E., Lowman D.W. (2008). Differential high-affinity interaction of dectin-1 with natural or synthetic glucans is dependent upon primary structure and is influenced by polymer chain length and side-chain branching. J. Pharmacol. Exp. Ther..

[38] Nelson T.E., Lewis B.A. (1974). Separation and characterization of the soluble and insoluble components of insoluble laminaran. Carbohydr. Res..

[39] Viola R., Nyvall P., Pedersén M. (2001). The unique features of starch metabolism in red algae. Proc. Biol. Sci..

[40] Amimi A., Mouradi A., Givernaud T., Chiadmi N., Lahaye M. (2001). Structural analysis of Gigartina pistillata carrageenans (Gigartinaceae, Rhodophyta). Carbohyd. Res..

[41] Running C.A., Falshaw R., Janaswamy S. (2012). Trivalent iron induced gelation in lambda-carrageenan. Carbohyd. Polym..

[42] Manuhara G.J., Praseptiangga D., Riyanto R.A. (2016). Extraction and Characterization of Refined K-carrageenan of Red Algae [*Kappaphycus Alvarezii* (Doty ex P.C. Silva, 1996)] Originated from Karimun Jawa Islands. Aquat. Procedia.

[43] Lebbar S., Fanuel M., Le Gall S., Falourd X., Ropartz D., Bressollier P., Gloaguen V., Faugeron-Girard C. (2018). Agar Extraction By-Products from *Gelidium sesquipedale* as a Source of Glycerol-Galactosides. Molecules.

[44] Lee W.-K., Lim Y.-Y., Leow A.T.-C., Namasivayam P., Ong Abdullah J., Ho C.-L. (2017). Biosynthesis of agar in red seaweeds: A review. Carbohyd. Polym..

[45] Leliaert F., Smith D.R., Moreau H., Herron M.D., Verbruggen H., Delwiche C.F., De Clerck O. (2012). Phylogeny and Molecular Evolution of the Green Algae. Crit. Rev. Plant. Sci..

[46] Sardari R.R.R., Nordberg Karlsson E. (2018). Marine Poly- and Oligosaccharides as Prebiotics. J. Agric. Food Chem..

[47] Wichard T., Charrier B., Mineur F., Bothwell J.H., Clerck O.D., Coates J.C. (2015). The green seaweed Ulva: A model system to study morphogenesis. Front. Plant. Sci..

[48] Mine I., Menzel D., Okuda K. (2008). Morphogenesis in giant-celled algae. Int. Rev. Cell Mol. Biol..

[49] Cocquyt E., Verbruggen H., Leliaert F., De Clerck O. (2010). Evolution and Cytological Diversification of the Green Seaweeds (Ulvophyceae). Mol. Biol. Evol..

[50] Cho M.L., You S.G., Kim S.-K. (2015). Sulfated polysaccharides from green seaweeds. Springer Handbook of Marine Biotechnology.

[51] Zhang M., Zhao C., Shao Q., Yang Z., Zhang X., Xu X., Hassan M. (2019). Determination of water content in corn stover silage using near-infrared spectroscopy. Int. J. Agric. Biol. Eng..

[52] Maneein S., Milledge J.J., Vejby Nielsen B., Harvey P. (2018). A Review of Seaweed Pre-Treatment Methods for Enhanced Biofuel Production by Anaerobic Digestion or Fermentation. Fermentation.

[53] Zhao C., Cao Y., Ma Z., Shao Q. (2017). Optimization of liquid ammonia pretreatment conditions for maximizing sugar release from giant reed (*Arundo donax* L.). Biomass Bioenerg..

[54] Kadam S.U., Álvares C., Twari B.K., O’Donnell C.P., Tiwari B.K., Troy D.J. (2015). Extraction of biomolecules from seaweeds. Seaweed Sustainability—Food and Non-Food Applications.

[55] Sosa-Hernández J.E., Escobedo-Avellaneda Z., Iqbal H.M.N., Welti-Chanes J. (2018). State-of-the-Art Extraction Methodologies for Bioactive Compounds from Algal Biome to Meet Bio-Economy Challenges and Opportunities. Molecules.

[56] Shiroma R., Uechi S., Taira T., Ishihara M., Tawata S., Tako M. (2003). Isolation and Characterization of Fucoidan from *Hizikia fusiformis* (Hijiki). J. Appl. Glycosci..

[57] Kim W.-J., Kim S.-M., Kim H.G., Oh H.-R., Lee K.-B., Lee Y.-K., Park Y.-I. (2007). Purification and Anticoagulant Activity of a Fucoidan from Korean *Undaria pinnatifida* Sporophyll. Algae.

[58] Hahn T., Lang S., Ulber R., Muffler K. (2012). Novel procedures for the extraction of fucoidan from brown algae. Process. Biochem..

[59] Wang C.-Y., Chen Y.-C. (2016). Extraction and characterization of fucoidan from six brown macroalgae. J. Mar. Sci. Technol..

[60] Liu X., Liu B., Wei X.L., Sun Z.L., Wang C.Y. (2016). Extraction, Fractionation, and Chemical Characterisation of Fucoidans from the Brown Seaweed *Sargassum pallidum*. Czech. J. Food Sci..

[61] Khalil H., Lai T., Tye Y., Rizal S., Chong E., Yap S., Hamzah A., Fazita M., Paridah M. (2018). A review of extractions of seaweed hydrocolloids: Properties and applications. Express Polym. Lett..

[62] Li C., Wang C., Wang S., Qian G., Zhu Q., Liu Y., Wang W. (2013). Optimization of Ultrasonic-assisted Extraction Technology of Sargassum fusiforme Polysaccharides and Evaluation of Their Antioxidant Activity. Food Sci. Technol. Res..

[63] Rafiquzzaman S.M., Rahman A., Kong I.-S. (2017). Ultrasonic-Assisted Extraction of Carrageenan. Seaweed Polysacch..

[64] Rodriguez-Jasso R.M., Mussatto S.I., Pastrana L., Aguilar C.N., Teixeira J.A. (2011). Microwave-assisted extraction of sulfated polysaccharides (fucoidan) from brown seaweed. Carbohyd. Polym..

[65] Yuan Y., Macquarrie D. (2015). Microwave assisted extraction of sulfated polysaccharides (fucoidan) from *Ascophyllum nodosum* and its antioxidant activity. Carbohyd. Polym..

[66] Lakmal H.H.C., Lee J.H., Jeon Y.J., Ramawat K.G., Mérillon J.-M. (2015). Enzyme-Assisted Extraction of a Marine Algal Polysaccharide, Fucoidan and Bioactivities. Polysaccharides - Bioactivity and Biotechnology.

[67] Aðalbjörnsson B.V., Jónsdóttir R. (2015). Enzyme-Enhanced Extraction of Antioxidant Ingredients from Algae. Natural Products from Marine Algae—Methods in Molecular Biology.

[68] Habeebullah S.F.K., Alagarsamy S., Sattari Z., Al-Haddad S., Fakhraldeen S., Al-Ghunaim A., Al-Yamani F. (2019). Enzyme-assisted extraction of bioactive compounds from brown seaweeds and characterization. J. Appl. Phycol..

[69] Puri M., Sharma D., Barrow C.J. (2012). Enzyme-assisted extracted of bioactives from plants. Trends Biotechnol..

[70] Michalak I., Górka B., Wieczorek P.P., Rój E., Lipok J., Łeska B., Messayasz B., Wilk R., Schroeder G., Dobrzy’nska-Inger A. (2016). Supercriticalfluid extraction of algae enhances levels ofbiologically active compounds promoting plant growth. Eur. J. Phycol..

[71] Messyasz B., Michalak I., Łęska B., Schroeder G., Górka B., Korzeniowska K., Lipok J., Wieczorek P., Rój E., Wilk R. (2018). Valuable natural products from marine and freshwater macroalgae obtained from supercritical fluid extracts. J. Appl. Phycol..

[72] Espinosa-Pardo F.A., Martinez J., Martinez-Corre H.A. (2014). Extraction of bioactive compounds from peach palm pulp (Bactris gasipaes) using supercritical CO_2_. J. Supercrit. Fluid..

[73] Rioux L.-E., Turgeon S.L., Beaulieu M. (2007). Characterization of polysaccharides extracted from brown seaweeds. Carbohyd. Polym..

[74] Rani V., Shakila R.J., Jawahar P., Srinivasan A. (2017). Influence of Species, Geographic Location, Seasonal Variation and Extraction Method on the Fucoidan Yield of the Brown Seaweeds of Gulf of Mannar, India. Indian J. Pharm. Sci..

[75] Ale M.T., Meyer A.S. (2013). Fucoidans from brown seaweeds: an update on structures, extraction techniques and use of enzymes as tools for structural elucidation. RSC Adv..

[76] Kimica About Alginate—Manufacturing Process. https://kimica-algin.com/alginate/process/.

[77] McHugh D.J. (2003). Alginate. A Guide to the Seaweed Industry.

[78] Mazumder A., Løvstad Holdt S., De Francisci D., Alvarado-Morales M., Mishra H.N., Angelidaki I. (2016). Extraction of alginate from *Sargassum muticum*: process optimization and study of its functional activities. J. Appl. Phycol..

[79] Fertah M., Belfkira A., Dahmane E.M., Taourirte M., Brouillette F. (2017). Extraction and characterization of sodium alginate from Moroccan *Laminaria digitata* brown seaweed. Arab. J. Chem..

[80] Alboofetileh M., Rezaei M., Tabarsa M., You S. (2018). Ultrasound-assisted extraction of sulfated polysaccharide from *Nizamuddinia zanardinii*: Process optimization, structural characterization, and biological properties. J. Food Process. Eng..

[81] Hmelkov A.B., Zvyagintseva T.N., Shevchenko N.M., Rasin A.B., Ermakova S.P. (2018). Ultrasound-assisted extraction of polysaccharides from brown alga *Fucus evanescens*. Structure and biological activity of the new fucoidan fractions. J. Appl. Phycol..

[82] Alboofetileh M., Rezaei M., Tabarsa M. (2019). Enzyme-assisted extraction of *Nizamuddinia zanardinii* for the recovery of sulfated polysaccharides with anticancer and immune-enhancing activities. J. Appl. Phycol..

[83] Je J.-Y., Park P.-J., Kim E.-K., Park J.-S., Yoon H.-D., Kim K.-R., Ahn C.-B. (2009). Antioxidant activity of enzymatic extracts from the brown seaweed *Undaria pinnatifida* by electron spin resonance spectroscopy. LWT-Food Sci. Technol..

[84] Men’shova R.V., Lepeshkin F.D., Ermakova S.P., Pokrovskii O.I., Zvyagintseva T.N. (2013). Effect of pretreatment conditions of brown algae by supercritical fluids on yield and structural characteristics of fucoidans. Chem. Nat. Compd..

[85] Youssouf L., Lallemand L., Giraud P., Soulé F., Bhaw-Luximon A., Meilhac O., Lefèbvre D’Hellencourt C., Jhurry D., Couprie J. (2017). Ultrasound-assisted extraction and structural characterization by NMR of alginates and carrageenans from seaweeds. Carbohyd. Polym..

[86] Li H., Yu X., Jin Y., Zhang W., Liu Y. (2008). Development of an eco-friendly agar extraction technique from the red seaweed *Gracilaria lemaneiformis*. Bioresour. Technol..

[87] Luz Arvizu-Higuera D., Rodriguez-Montesinos Y.E., Murillo-Alvarez J.I., Munoz-Ochoa M., Hernandez-Carmona G., Borowitzka M.A., Critchley A.T., Kraan S., Peters A., Sjøtun K., Notoya M. (2007). Effect of alkali treatment time and extraction time on agar from *Gracilaria vermiculophylla*. Nineteenth International Seaweed Symposium.

[88] Distantina S., Wiratni W., Fahrurrozi M., Rochmadi R. (2011). Carrageenan properties extracted from *Eucheuma cottonii*, Indonesia. World Acad. Sci. Eng. Technol..

[89] Tuvikene R., Truus K., Vaher M., Kailas T., Martin G., Kersen P. (2006). Extraction and quantification of hybrid carrageenans from the biomass of the red algae *Furcellaria lumbricalis* and *Coccotylus truncatus*. Proc. Estonian Acad. Sci. Chem..

[90] Blanco-Pascual N., Alemán A., Gómez-Guillén M.C., Montero M.P. (2014). Enzyme-assisted extraction of κ/ι-hybrid carrageenan from *Mastocarpus stellatus* for obtaining bioactive ingredients and their application for edible active film development. Food Funct..

[91] Mustapha S., Chandar H., Abidin Z.Z., Saghravani R., Harun M.Y. (2011). Production of semi-refined carrageenan from Eucheuma cotonii. J. Sci. Ind. Res..

[92] Freile-Pelegrín Y., Robledo D. (2008). Carrageenan of *Eucheuma isiforme* (Solieriaceae, Rhodophyta) from Nicaragua. J. Appl. Phycol..

[93] Webber V., Matos de Carvalho S., Ogliari P.J., Hayashi L., Barreto P.L.M. (2012). Optimization of the extraction of carrageenan from *Kappaphycus alvarezii* using response surface methodology. Food Sci. Technol..

[94] Bono A., Anisuzzaman S.M., Ding O.W. (2014). Effect of process conditions on the gel viscosity and gel strength of semi-refined carrageenan (SRC) produced from seaweed (*Kappaphycus alvarezii*). J. King Saud Univ. Sci..

[95] Hernández-Carmona G., Freile-Pelegrín Y., Hernández-Garibay E., Domínguez H. (2013). Conventional and alternative technologies for the extraction of algal polysaccharides. Functional Ingredients from Algae for Foods and Nutraceuticals.

[96] Heriyanto H., Kustiningsih I., Sari D.K. (2018). The effect of temperature and time of extraction on the quality of Semi Refined Carrageenan (SRC). MATEC Web Conf..

[97] Rao A.V., Bekheet I.A. (1976). Preparation of agar-agar from the red seaweed *Pterocladia capillacea* off the coast of Alexandria, Egypt. Appl. Environ. Microbiol..

[98] Torres M.D., Flórez-Fernández N., Domínguez H. (2019). Integral Utilization of Read Seaweed for Bioactive Production. Mar. Drugs.

[99] Circuncisão A.R., Catarino M.D., Cardoso S.M., Silva A.M.S. (2018). Minerals from Macroalgae Origin: Health Benefits and Risks for Consumer. Mar. Drugs.

[100] Mišurcová L., Machů L., Orsavová J. (2011). Seaweed minerals as nutraceuticals. Adv. Food Nutr. Res..

[101] Wei M., Wanibuchi H., Morimura K., Iwai S., Yoshida K., Endo G., Nakae D., Fukushima S. (2002). Carcinogenicity of dimethylarsinic acid in male F344 rats and genetic alterations in induced urinary bladder tumors. Carcinogenesis.

[102] Yamamoto S., Konishi Y., Matsuda T., Murai T., Shibata M.A., Matsui-Yuasa I., Otani S., Kuroda K., Endo G., Fukushima S. (1995). Cancer induction by an organic arsenic compound, dimethylarsinic acid (cacodylic acid), in F344/DuCrj rats after pretreatment with five carcinogens. Cancer Res..

[103] van Weelden G., Bobinski M., Okła K., van Weelden W.J., Romano A., Pijnenborg J.M.A. (2018). Fucoidan Structure and Activity in Relation to Anti-Cancer Mechanisms. Mar. Drugs.

[104] Helbert W. (2017). Marine Polysaccharide Sulfatases. Front. Mar. Sci..

[105] Hanson S.R., Best M.D., Wong C.-H. (2004). Sulfatases: Structure, Mechanism, Biological Activity, Inhibition, and Synthetic Utility. Angew. Chem. Int. Ed. Engl..

[106] Ertesvåg H. (2015). Alginate-modifying enzymes: biological roles and biotechnological uses. Front Microbiol..

[107] Szekalska M., Puciłowska A., Szymańska E., Ciosek P., Winnicka K. (2016). Alginate: Current Use and Future Perspectives in Pharmaceutical and Biomedical Applications. Int. J. Polym. Sci..

[108] Holdt S.L., Kraan S. (2011). Bioactive compounds in seaweed: functional food applications and legislation. J. Appl. Phycol..

[109] Becker S., Scheffel A., Polz M.F., Hehemann J.-H. (2017). Accurate Quantification of Laminarin in Marine Organic Matter with Enzymes from Marine Microbes. Appl. Environ. Microbiol.

[110] Brown G.D., Taylor P.R., Reid D.M., Willment J.A., Williams D.L., Martinez-Pomares L., Gordon S. (2002). Dectin-1 is a major beta-glucan receptor on macrophages. J. Exp. Med..

[111] Taylor P.R., Brown G.D., Reid D.M., Willment J.A., Martinez-Pomares L., Gordon S., Wong S.Y.C. (2002). The β-glucan receptor, dectin-1, is predominantly expressed on the surface of cells of the monocyte/macrophage and neutrophil lineages. J. Immunol..

[112] Pang Z., Otaka K., Maoka T., Hidaka K., Ishikima S., Oda M., Ohnishi M. (2005). Structure of β-Glucan Oligomer from Laminarin and Its Effect on Human Monocytes to Inhibit the Proliferation of U937 Cells. Biosci. Biotechnol. Biochem..

[113] Dobruchowska J.M., Jonsson J.O., Fridjonsson O.H., Aevarsson A., Kristjansson J.K., Altenbuchner J., Watzlawick H., Gerwig G.J., Dijkhuizen L., Kamerling J.P. (2016). Modification of linear (β1→3)-linked gluco-oligosaccharides with a novel recombinant β-glucosyltransferase (trans-β-glucosidase) enzyme from *Bradyrhizobium diazoefficiens*. Glycobiology.

[114] Jonsson-Wheat J.O., Hreggvidsson G.O., Fridjonsson O.H., Dobruchowska J.M., Kamerling J.P. (2014). Glucan Branching Enzymes and Their Methods of Use.

[115] Hreggviðsson G.H., Dobruchowska J.M., Fridjonsson O.H., Jonsson J.O., Gerwig G.J., Aevarsson A., Kristjansson J.K., Curti D., Redgwell R.R., Hansen C.-E. (2011). Exploring novel non-Leloir β-glucosyltransferases from proteobacteria for modifying linear (β1 → 3)-linked gluco-oligosaccharide chains. Glycobiology.

[116] Michel G., Czjzek M., Trincone A. (2013). Polysaccharide-degrading enzymes from marine bacteria. Marine Enzymes for Biocatalysis—Sources, Biocatalytic Characteristics and Bioprocesses of Marine Enzymes.

[117] Barbeyron T., Henrissat B., Kloareg B. (1994). The gene encoding the kappa-carrageenase of *Alteromonas carrageenovora* is related to β-1,3-1,4-glucanases. Gene.

[118] Barbeyron T., Gerard A., Potin P., Henrissat B., Kloareg B. (1998). The kappa-carrageenase of the marine bacterium *Cytophaga drobachiensis*. Structural and phylogenetic relationships within family-16 glycoside hydrolases. Mol. Biol. Evol..

[119] Ohta Y., Hatada Y. (2006). A Novel Enzyme, λ-Carrageenase, Isolated from a Deep-Sea Bacterium. J. Biochem..

[120] Fu X.T., Kim S.M. (2010). Agarase: review of major sources, categories, purification method, enzyme characteristics and applications. Mar. Drugs.

[121] Ohta Y., Hatada Y., Miyazaki M., Nogi Y., Ito S., Horikoshi K. (2005). Purification and Characterization of a Novel α-Agarase from a Thalassomonas sp.. Curr. Microbiol..

[122] Rochas C., Potin P., Kloareg B. (1994). NMR spectroscopic investigation of agarose oligomers produced by an α-agarase. Carbohyd. Res..

[123] Konasani V.R., Jin C., Karlsson N.G., Albers E. (2018). A novel ulvan lyase family with broad-spectrum activity from the ulvan utilisation loci of *Formosa agariphila* KMM 3901. Sci. Rep..

[124] Sharma S.D., Pati M., Nayak L. (2016). Uses of Seaweed and Its Application to Human Welfare: A Review. Int. J. Pharm. Pharm. Sci..

[125] Makkar H., Tran G., Heuzé V., Giger-Reverdin S., Lessire M., Lebas F., Ankers P. (2015). Seaweeds for livestock diets: A review. Anim. Feed Sci. Technol..

[126] Delaney A., Frangoudes K., Ii S.A., Fleurence J., Levine I. (2016). Society and Seaweed: Understanding the Past and Present. Seaweed in Health and Disease Prevention.

[127] Mouritsen O.G. (2013). Seaweeds: Edible, Available & Sustainable.

[128] Hamid N., Ma Q., Boulom S., Liu T., Zheng Z., Balbas J., Robertson J., Tiwari B.K., Troy D.J. (2015). Seaweed minor constituents. Seaweed Sustainability.

[129] Venkatraman K.L., Mehta A. (2019). Health Benefits and Pharmacological Effects of Porphyra Species. Plant. Food Hum. Nutr..

[130] van der Weele C., Feindt P., van der Goot A.J., van Mierlo B., van Boekel M. (2019). Meat alternatives: An integrative comparison. Trends Food Sci. Technol..

[131] Ganesan A.R., Tiwari U., Rajauria G. (2019). Seaweed nutraceuticals and their therapeutic role in disease prevention. Food Sci. Hum. Wellness.

[132] Wan A.H.L., Davies S.J., Soler-Vila A., Fitzgerald R., Johnson M.P. (2019). Macroalgae as a sustainable aquafeed ingredient. Rev. Aquacult..

[133] Shiroma R., Konishi T., Uechi S., Tako M. (2008). Structural Study of Fucoidan from the Brown Seaweed *Hizikia fusiformis*. Food Sci. Technol. Res..

[134] Kadena K., Tomori M., Iha M., Nagamine T. (2018). Absorption Study of Mozuku Fucoidan in Japanese Volunteers. Mar. Drugs.

[135] Hsu H.-Y., Hwang P.-A. (2019). Clinical applications of fucoidan in translational medicine for adjuvant cancer therapy. Clin. Trans. Med..

[136] Atashrazm F., Lowenthal R.M., Woods G.M., Holloway A.F., Dickinson J.L. (2015). Fucoidan and Cancer: A Multifunctional Molecule with Anti-Tumor Potential. Mar. Drugs.

[137] Lee K.Y., Mooney D.J. (2012). Alginate: Properties and biomedical applications. Prog. Polym. Sci..

[138] Pongsawatmanit R., Ikeda S., Miyawaki O. (1998). Effect of temperature on viscoelastic properties of aqueous alginate solutions. Thai J. Agric. Sci..

[139] Senturk Parreidt T., Müller K., Schmid M. (2018). Alginate-Based Edible Films and Coatings for Food Packaging Applications. Foods.

[140] Qin Y., Jiang J., Zhao L., Zhang J., Wang F., Grumezescu A., Butu A. (2018). Applications of Alginate as a Functional Food Ingredient. Biopolymers for Food Design.

[141] Jensen M.G., Kristensen M., Astrup A. (2012). Effect of alginate supplementation on weight loss in obese subjects completing a 12-wk energy-restricted diet: A randomized controlled trial. Am. J. Clin. Nutr..

[142] Houghton D., Wilcox M.D., Brownlee I.A., Chater P.I., Seal C.J., Pearson J.P. (2019). Acceptability of alginate enriched bread and its effect on fat digestion in humans. Food Hydrocoll..

[143] Odunsi S.T., Vázquez-Roque M.I., Camilleri M., Papathanasopoulos A., Clark M.M., Wodrich L., Lempke M., McKinzie S., Ryks M., Burton D. (2010). Effect of Alginate on Satiation, Appetite, Gastric Function, and Selected Gut Satiety Hormones in Overweight and Obesity. Obesity.

[144] Mantovani M.S., Bellini M.F., Angeli J.P., Oliveira R.J., Silva A.F., Ribeiro L.R. (2008). β-Glucans in promoting health: Prevention against mutation and cancer. Mutat. Res..

[145] Aziz A., Poinssot B., Daire X., Adrian M., Bézier A., Lambert B., Joubert J.M., Pugin A. (2003). Laminarin Elicits Defense Responses in Grapevine and Induces Protection Against *Botrytis cinerea* and *Plasmopara viticola*. Mol. Plant. Microbe Interact..

[146] UPL Improve Overall Plant Health with Vacciplant®. https://us.uplonline.com/product-details/vacciplant.

[147] Seong H., Bae J.-H., Seo J.S., Kim S.-A., Kim T.-J., Han N.S. (2019). Comparative analysis of prebiotic effects of seaweed polysaccharides laminaran, porphyran, and ulvan using in vitro human fecal fermentation. J. Funct. Foods.

[148] Devillé C., Gharbi M., Dandrifosse G., Peulen O. (2007). Study on the effects of laminaran, a polysaccharide from seaweed, on gut characteristics. J. Sci. Food Agric..

[149] Zekovic D.B., Kwiatkowski S., Vrvic M.M., Jakovljevic D., Moran C.A. (2005). Natural and modified (1→3)-β-D-glucans in health promotion and disease alleviation. Crit. Rev. Biotechnol..

[150] Dalonso N., Goldman G.H., Gernm R.M. (2015). β-(1→3),(1→6)-Glucans: Medicinal activities, characterization, biosynthesis and new horizons. Appl. Microbiol. Biotechnol..

[151] Park H.-K., Kim I.-H., Kim J., Nam T.-J. (2013). Induction of apoptosis and the regulation of ErbB signaling by laminarin in HT-29 human colon cancer cells. Int. J. Mol. Med..

[152] Bonfim-Mendonça P.S., Capoci I., Tobaldini-Valerio F.K., Negri M., Svidzinski T. (2017). Overview of β-Glucans from Laminaria spp.: Immunomodulation properties and applications on biologic models. Int. J. Mol. Sci..

[153] Stier H., Ebbeskotte V., Gruenwald J. (2014). Immune-modulatory effects of dietary Yeast Beta-1,3/1,6-D-glucan. Nutr. J..

[154] Distantina S., Rochmadi R., Fahrurrozi M., Wiratni W. (2013). Synthesis of Hydrogel Film Based on Carrageenan Extracted from *Kappaphycus alvarezii*. Mod. Appl. Sci..

[155] Qin Y., Qin Y. (2018). Seaweed Hydrocolloids as Thickening, Gelling, and Emulsifying Agents in Functional Food Products. Bioactive Seaweeds for Food Applications.

[156] Pangestuti R., Kim S.-K., Kim S.-K. (2014). Biological Activities of Carrageenan. Advances in Food and Nutrition Research.

[157] Carlucci M.J., Scolaro L.A., Noseda M.D., Cerezo A.S., Damonte E.B. (2004). Protective effect of a natural carrageenan on genital herpes simplex virus infection in mice. Antivir. Res..

[158] Eccles R., Meier C., Jawad M., Weinmüllner R., Grassauer A., Prieschl-Grassauer E. (2010). Efficacy and safety of an antiviral Iota-Carrageenan nasal spray: A randomized, double-blind, placebo-controlled exploratory study in volunteers with early symptoms of the common cold. Respir. Res..

[159] Panlasigui L., Baello O., Dimatangal J., Dumelod B. (2003). Blood cholesterol and lipid-lowering effects of carrageenan on human volunteers. Asia. Pac. J. Clin. Nutr..

[160] Haijin M., Xiaolu J., Huashi G. (2003). A κ-carrageenan derived oligosaccharide prepared by enzymatic degradation containing anti-tumor activity. J. Appl. Phycol..

[161] Zhou G., Sheng W., Yao W., Wang C. (2006). Effect of low molecular λ-carrageenan from Chondrus ocellatus on antitumor H-22 activity of 5-Fu. Pharmacol. Res..

[162] Zhou G., Sun Y., Xin H., Zhang Y., Li Z., Xu Z. (2004). In vivo antitumor and immunomodulation activities of different molecular weight lambda-carrageenans from Chondrus ocellatus. Pharmacol. Res..

[163] Sokolova E.V., Barabanova A.O., Homenko V.A., Solov’eva T.F., Bogdanovich R.N., Yermak I.M. (2011). In Vitro and Ex Vivo Studies of Antioxidant Activity of Carrageenans, Sulfated Polysaccharides from Red Algae. Bull. Exp. Biol. Med..

[164] Rocha de Souza M.C., Marques C.T., Guerra Dore C.M., Ferreira da Silva F.R., Oliveira Rocha H.A., Leite E.L. (2007). Antioxidant activities of sulfated polysaccharides from brown and red seaweeds. J. Appl. Phycol..

[165] Hu X., Jiang X., Aubree E., Boulenguer P., Critchley A.T. (2006). Preparation and In Vivo. Antitumor Activity of κ-Carrageenan Oligosaccharides. Pharm. Biol..

[166] Yuan H., Zhang W., Li X., Lü X., Li N., Gao X., Song J. (2005). Preparation and in vitro antioxidant activity of κ-carrageenan oligosaccharides and their oversulfated, acetylated, and phosphorylated derivatives. Carbohyd. Res..

[167] Xu L., Yao Z., Wu H., Wang F., Zhang S. (2012). The immune regulation of κ-carrageenan oligosaccharide and its desulfated derivatives on LPS-activated microglial cells. Neurochem. Int..

[168] Chauhan P.S., Saxena A. (2016). Bacterial carrageenases: An overview of production and biotechnological applications. 3 Biotech..

[169] Li L., Ni R., Shao Y., Mao S. (2014). Carrageenan and its applications in drug delivery. Carbohyd. Polym..

[170] Lokhande G., Carrow J.K., Thakur T., Xavier J.R., Parani M., Bayless K.J., Gaharwar A.K. (2018). Nanoengineered injectable hydrogels for wound healing application. Acta Biomat..

[171] Vera J., Castro J., Gonzalez A., Moenne A. (2011). Seaweed polysaccharides and derived oligosaccharides stimulate defense responses and protection against pathogens in plants. Mar. Drugs.

[172] Izydorczyk M., Cui S., Wang Q., Cui S.W. (2005). Polysaccharide Gums: Structures, Functional Properties, and Applications. Food Carbohydrates: Chemistry, Physical Properties and Applications.

[173] Marcus J.B., Marcus J.B. (2013). Food Science Basics: Healthy Cooking and Baking Demystified: The Science behind Healthy Foods, Cooking and Baking. Culinary Nutrition.

[174] Armisén R., Gaiatas F., Phillips G.O., Williams P.A. (2009). Agar. Handbook of Hydrocolloids.

[175] Serwer P. (1983). Agarose gels: Properties and use for electrophoresis. Electrophoresis.

[176] Armisén R. (1991). Agar and agarose biotechnological applications. Hydrobiologia.

[177] Ream J.A., Lewis L.K., Lewis K.A., Kurien B.T., Scofield R.H. (2019). Horizontal Agarose Gel Mobility Shift Assay for Protein-RNA Complexes. Electrophoretic Separation of Proteins: Methods and Protocols.

[178] Aurelien F., Jon C., Steffen L., Esther K., Simon T., Maziar M., Ralf T., Shastri V.P. (2013). Polysaccharide hydrogels with tunable stiffness and provasculogenic properties via α-helix to β-sheet switch in secondary structure. Proc. Natl. Acad. Sci. USA.

[179] Forget A., Pique R.A., Ahmadi V., Lu¨deke S., Shastri V.P. (2015). Mechanically Tailored Agarose Hydrogels through Molecular Alloying with β-Sheet Polysaccharides. Macromol. Rapid Commun..

[180] Qi H., Zhao T., Zhang Q., Li Z., Zhao Z., Xing R. (2005). Antioxidant activity of different molecular weight sulfated polysaccharides from *Ulva pertusa* Kjellm (Chlorophyta). J. Appl. Phycol..

[181] Karnjanapratum S., You S.G. (2011). Molecular characteristics of sulfated polysaccharides from *Monostroma nitidum* and their in vitro anticancer and immunomodulatory activities. Int. J. Biol. Macromol..

[182] Leiro J.M., Castro R., Arranz J.A., Lamas J. (2007). Immunomodulating activities of acidic sulphated polysaccharides obtained from the seaweed *Ulva rigida* C. Agardh. Int. Immunopharmacol..

[183] Qi H., Huang L., Liu X., Liu D., Zhang Q., Liu S. (2012). Antihyperlipidemic activity of high sulfate content derivative of polysaccharide extracted from *Ulva pertusa* (Chlorophyta). Carbohydr. Polym..

[184] Mao W.J., Fang F., Li H.Y., Qi X.H., Sun H.H., Chen Y., Guo S.D. (2008). Heparinoid-active two sulfated polysaccharides isolated from marine green algae *Monostroma nitidum*. Carbohydr. Polym..

